# Inhibition of Sterol Biosynthesis Alters Tubulin Association with Detergent-Insoluble Membranes and Affects Microtubule Organization in Pollen Tubes of *Nicotiana tabacum* L.

**DOI:** 10.3390/plants14243845

**Published:** 2025-12-17

**Authors:** Elisabetta Onelli, Lilly Maneta-Peyret, Patrick Moreau, Nadia Stroppa, Valeria Berno, Eugenia Cammarota, Marco Caccianiga, Luca Gianfranceschi, Alessandra Moscatelli

**Affiliations:** 1 Dipartimento di Bioscienze, Università degli Studi di Milano, Via Celoria 26, 20133 Milan, Italy; elisabetta.onelli@unimi.it (E.O.); nadiastroppa.ns@gmail.com (N.S.); marco.caccianiga@unimi.it (M.C.); luca.gianfranceschi@unimi.it (L.G.); 2 CNRS, Laboratoire de Biogenèse Membranaire, University of Bordeaux, UMR 5200, 71 Avenue Edouard Bourlaux, 33140 Villenave d’Ornon, France; lilly.maneta-peyret@u-bordeaux.fr (L.M.-P.); patrick.moreau@u-bordeaux.fr (P.M.); 3 ALEMBIC Advanced Light and Electron Microscopy BioImaging Center, San Raffaele Scientific Institute, DIBIT 1, Via Olgettina 58, 20132 Milan, Italy; berno.valeria@hsr.it (V.B.); eugenia.cammarota@fht.org (E.C.)

**Keywords:** pollen tube, microtubules, sterols, lipid rafts

## Abstract

Pollen tube growth entails complex molecular interactions between the cytoskeletal apparatus and membrane trafficking. Tip growth involves polarized distribution of proteins and lipids along the plasma membrane, including liquid-ordered microdomains, rich in sterols and sphingolipids (lipid rafts), in the apical/subapical region of tobacco pollen tubes. Intriguingly, biochemical characterization of detergent-insoluble membranes purified from tobacco pollen tubes revealed the presence of both actin and tubulin. Here, we report that inhibition of sterol biosynthesis altered lipid rafts and lowered the association of tubulin with detergent-insoluble membranes. Our results showed that sterol depletion increased the number of microtubules in the subapical region, altered microtubule distribution and affected microtubule bundling activity. Oryzalin washout experiments also suggested that lipid-ordered domains could play a role in regulating microtubule nucleation/regrowth.

## 1. Introduction

Pollen tubes are safe routes to convey sperm cells to the embryo sac for double fertilization. The efficiency of pollen tube growth is therefore a key issue for sexual reproduction in higher plants and for improving the genetic variability of species [1,2]. Pollen tube tip growth exploits complex molecular interactions to couple the polarized distribution of proteins and lipids along the plasma membrane (PM) with cytoskeleton dynamics and polarized exocytosis [3,4,5].

In the last 20 years, our understanding of the complexity of the PM has increased with the revision of the mosaic fluid model [6]. The revised model involves membrane nano- or micro-domains, enriched in sterols and sphingolipids (lipid rafts or liquid-ordered domains—Lo), which alternate with more fluid liquid-disordered domains (Ld) [7,8,9,10,11]. Lipid rafts give the PM a higher order of complexity, confining certain proteins preferentially in Lo domains as opposed to Ld domains, and thus helping compartmentalize different signaling pathways [12,13] and regulate polarized secretion [14,15].

Close lateral associations between sterols, sphingolipids and highly saturated phospholipids are the basis of the lipid raft purification strategy, which entails the non-ionic detergent insolubility of lipid rafts at low temperature [9,16,17,18,19]. It is therefore possible to isolate detergent-insoluble membranes (DIMs/lipid rafts) from different types of plant cells, including tobacco pollen tubes [20,21,22,23]. Biochemical characterization of DIMs and protein analysis by mass spectrometry has shown actin and tubulin in DIMs isolated from tobacco pollen tubes [23].

In pollen tubes, organelles and vesicles move by reverse-fountain cytoplasmic streaming along bundles of actin filaments (AFs), which extend in the cytoplasm up to about 10 µm from the apical PM [24,25,26]. Near the tip, cytoplasmic streaming ceases and the actin re-organizes to form an actin fringe, made up of short dynamic AFs, in the apex [27,28,29]. Actin fringe dynamics rely on a tip-localized signaling pathway involving ROP1, a member of the Rho-related small GTPase protein family. ROP1 and ROP-Interactive Crib motif-containing proteins (RIC1, 3, and 4), which couple Ca2+ fluxes with actin fringe dynamics and with polarized exocytosis [30,31,32,33,34], require integral lipid raft nanodomains [15,35,36].

Recent data showed that ROPs also regulate MT organization, acting on Interactor of Constitutive active ROP (ICR) partners [37,38]. The pollen tubes of angiosperms have an extensive system of microtubules (MTs), mainly localized in the cortex [39,40]. Several studies demonstrate that the integrity of the MT cytoskeleton is necessary to regulate vacuole positioning and entrance of the male germ unit into the tube [41,42]. More recent time-lapse experiments show that MTs control endocytosis in the tip region and trafficking of endocytic vesicles/endosomes to vacuoles [40,43,44].

Microtubules are polymers made up of α-/β-tubulin heterodimers that bind head-to-tail to form protofilaments; classically, 13 protofilaments associate laterally to form close, hollow tubes [45,46]. Microtubule polymers have dynamic instability, undergoing polymerization/depolymerization cycles [47,48,49], which depends on the particular composition of α- and β-tubulin isoforms [50,51] and their post-translational modifications [52,53]. These intrinsic properties of MTs modulate MT nucleation and bundling [54,55,56,57,58] and regulate MT interactions with the PM [59,60,61,62].

Despite these emerging insights into the role of MTs in membrane trafficking, little is known about the factors regulating the origin and geometry of the MT apparatus in pollen tubes [43]. Ultrastructural observations on *Nicotiana alata* pollen tubes after rapid freeze fixation and substitution have revealed close relationships between the cortical cytoskeleton and the PM. This cortical network comprised AFs, elements of endoplasmic reticulum and MTs that appeared to be extensively linked to the PM [63]. More recent studies identified two MT-binding polypeptides involved in cross-bridging cortical MTs to the PM in *Nicotiana tabacum* pollen tubes [64]. These structural connections suggest that the interaction of MTs with the PM could play a role in regulating MT organization; in this view, the lipid profile of the PM could play a role in regulating MT/PM contacts.

The presence of tubulin in DIMs, together with the localization of more dynamic MTs in the apex and shank [40], which parallels the polarized distribution of sterols and Lo domains in the PM [23], prompted us to investigate the contribution of lipid rafts to the organization of cortical MTs in growing pollen tubes of tobacco. As sterols are major factors for lipid partitioning [16,17,65,66] and for maintaining the integrity of lipid rafts [67], we designed an experimental approach involving inhibition of sterol biosynthesis.

We found that sterol depletion affected the association of tubulin with isolated DIMs, as well as the organization and recovery of MTs in oryzalin washout experiments in tobacco pollen tubes. Our results suggest that lipid rafts could be new actors in the complex mosaic of relationships that coordinate the nucleation, bundling and stability of MTs during pollen tube growth.

## 2. Results

### 2.1. Squalestatin Affects Sterol Biosynthesis and Delays Pollen Tube Emission

Squalestatin (Sq) has been widely employed to study the role of sterols and lipid rafts in evolutionarily distant cells such as neurons and pollen tubes [68,69,70]. It inhibits the enzyme squalene synthase that catalyzes the first reaction of the mevalonate-isoprenoid pathway, exclusively concerned with sterol biosynthesis, without affecting the production of farnesyl diphosphate involved in protein isoprenylation [71], which regulates the function of small GTPases of the RAS/RHO family [72,73,74].

**Figure 1:**
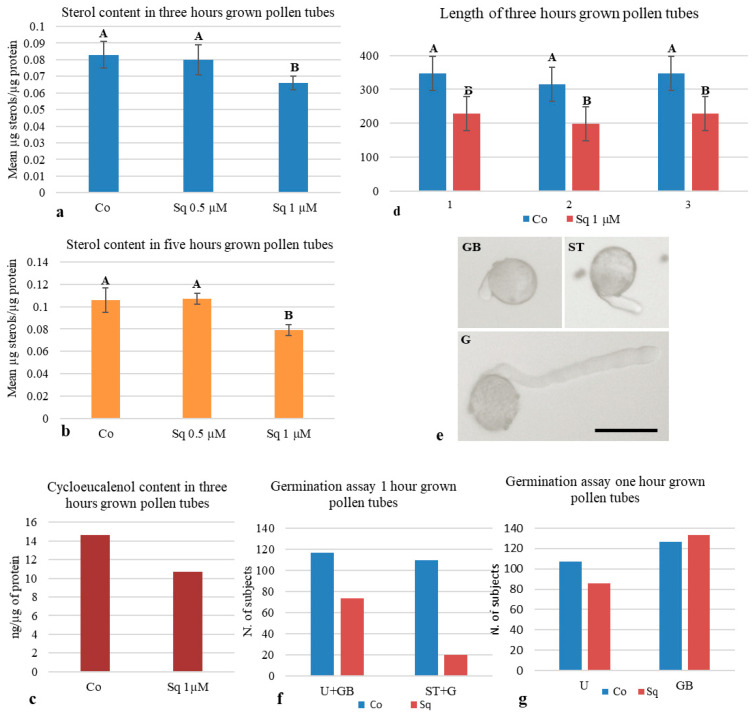
Effect of squalestatin on sterol content, pollen germination and pollen tube length. (**a**,**b**) Tobacco pollen was germinated with 0.5 and 1 µM Sq for 3 and 5 h. Sterol reduction was observed in pollen tubes germinated for 3 and 5 h with 1 µM Sq (one-way ANOVA and post hoc Tukey test, *p* = 0.02639 and *p* = 0.001125, respectively; *n* = 4), whereas 0.5 µM Sq did not inhibit sterol biosynthesis (one-way ANOVA and post hoc Tukey test, *p* > 0.05; *n* = 4). (**c**) Further analysis showed that 1 µM Sq inhibited de novo synthesis of cycloeucalenol after germination for 3 h (*n* = 2). (**d**) Pollen tube length measured by ImageJ software (version 2.9.0) after germination for 3 h decreased in Sq-treated pollen tubes (Student’s *t*-test; in the three experiments shown in (**d**); in each experiment, ≥110 pollen tubes were measured for each sample). (**e**–**g**) Germination assays were performed with and without Sq. The number of ungerminated pollen grains (U), pollen with germination bulges (GBs), short tubes (STs) or germinated tubes (Gs) were considered (**e**); scale bar = 50 µm. After germination for 1 h, the number of germinated pollen grains (ST + G) compared with ungerminated (U + GB) was significantly lower in Sq-treated samples (**f**); chi-square test (*p* < 0.0001). The chi-square test also showed that GB increased signicantly in the presence of Sq (**g**); chi-square test (*p* = 0.03). The histograms show data pooled from three experiments. Capital letters in the histograms indicate whether or not there are significant differences.

To verify the effect of Sq on sterol biosynthesis in tobacco pollen tubes, variations in sterol content were assessed as differences in the sterol/protein ratio in microsomes (P2, see below) isolated from pollen tubes grown in control medium and with 0.5 or 1 µM Sq for 3 and 5 h. A significant decrease in total sterols was observed in microsomes purified from pollen tubes grown in with 1 µM Sq for 3 and 5 h (Figure 1a,b, respectively).

No reduction in total sterols was observed when pollen tubes were grown with 0.5 µM Sq for the same times (Figure 1a,b), suggesting that 0.5 µM Sq was not sufficient to inhibit squalene synthase in tobacco pollen tubes, even at long incubation times. As the total sterol content decreased after incubation for 3 h with 1 µM Sq, these conditions were used in further experiments.

Recent studies showed changes in the lipid profile during pollen maturation in different angiosperms [69]; metabolomic analysis identified eight metabolic phases during tobacco pollen development and pollen tube growth [75]. During pollen germination and pollen tube growth, de novo synthesis of cycloeucalenol and an unidentified M 412 sterol was described [69,70,71,72,73,74,75]. To investigate the effect of Sq on cycloeucalenol, microsomes purified from control and treated pollen tubes were analyzed by GC-MS, and showed that the Sq treatment of pollen tubes decreased the amount of cycloeucalenol with respect to the control (Figure 1c).

To test the effects of inhibition of cycloeucalenol biosynthesis on pollen tube growth, pollen tube elongation was tracked in control and treated pollen tubes. Pollen tubes grown with 1 µM Sq for 3 h (Figure 1d and Figure S1A(a,b)) and 5 h (Figure S1B(a–c)) were significantly shorter than controls in three different experiments.

However, the pollen tubes did not show evident alterations in morphology (Figure S1A(a,b),B(b,c)). This observation suggests that the choice of inhibitor and its concentration did not induce tip swelling or depolarized growth [15,30,31], allowing for longer, more accurate observations.

In a previous study, detailed analysis of tube growth dynamics revealed that Sq induced a delay in tip distension without affecting the pollen tube growth rate [70]; therefore, we postulated that the decrease in pollen tube length could be due to a delay in pollen germination. To investigate this point, we performed in vitro germination assays. Tobacco pollen was germinated with or without 1 µM Sq and samples were obtained after 1 h. Specimens were observed by light microscopy for ungerminated pollen (U), pollen grains with germination bulges (GBs), pollen with short tubes (STs, with a pollen tube length of less than twice the pollen diameter) and germinated pollen (G, with a pollen tube length of more than twice the pollen diameter) (Figure 1e and Figure S1C(a,b)). The number of germinated pollen grains (ST + G) under control conditions was significantly greater than in Sq-treated samples after 1 h of incubation in three independent experiments (Figure 1f and Figure S1C(a,b)). Interestingly, the statistical analysis comparing U and GB revealed a significant increase in GB in Sq samples compared to the control. These data suggest that sterol depletion, and particularly the inhibition of cycloeucalenol biosynthesis, affected the early stages of pollen tube emission, thus determining a general decrease in pollen tube length.

### 2.2. Sterol Depletion Affects Interaction Between Tubulin and Detergent-Insoluble Membranes

Although tubulin is a soluble protein, when tobacco pollen tubes are homogenized, tubulin/tubulin oligomers or short MTs remain associated with cell membranes [23]. While the nature of this association remains unclear, we postulated that changes in the lipid profile could affect the interaction between tubulin/MTs and cell membranes.

**Figure 2:**
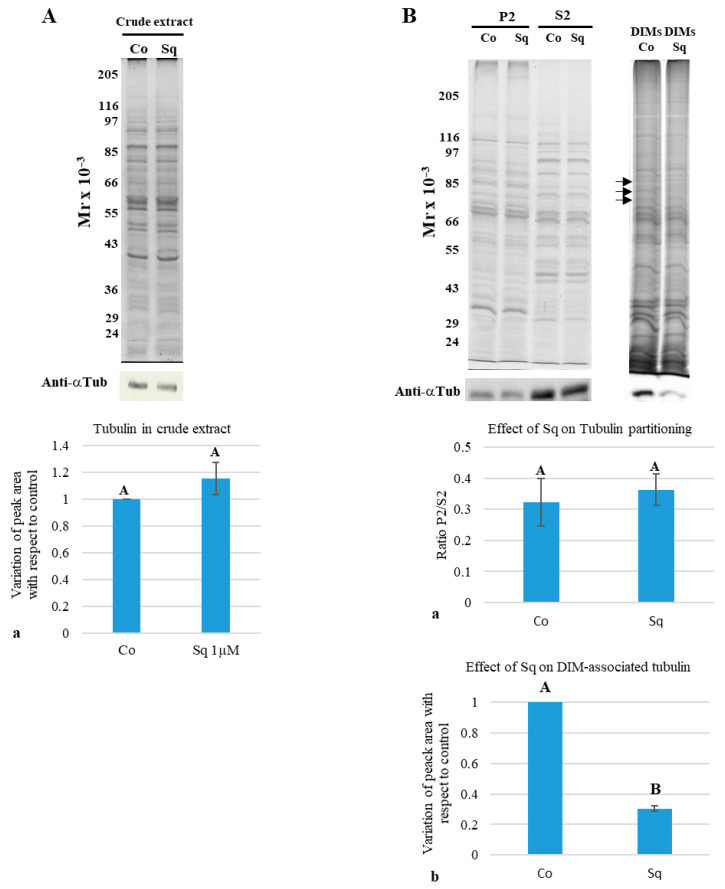
Tubulin partitioning between soluble and microsomal fractions, and between control and Sq-DIMs. (**A**) Electrophoretic analysis of crude extracts did not show any difference in polypeptide profile (crude extract, Coomassie blue-stained SDS-PAGE; 10 µg protein in each lane) between control and Sq-treated pollen tubes. Western blot of control and Sq-treated pollen tube crude extracts using a monoclonal anti-α-tubulin antibody (Anti-αTub) showed a similar amount of tubulin in the two samples ((**a**) Student’s *t*-test, *p* > 0.05; *n* = 5). (**B**) Electrophoretic analysis of microsomal (P2) and soluble fractions (S2) from pollen tubes grown with or without Sq did not show any differences in electrophoretic profile between control and Sq-treated samples (P2/S2, Coomassie blue-stained SDS-PAGE; 10 µg protein in each lane). Western blot analysis of P2/S2 samples from control and Sq-treated pollen tubes using a monoclonal anti-α-tubulin antibody showed that Sq depletion did not alter tubulin partitioning between P2 and S2 ((**a**) Student’s *t*-test, *p* > 0.05; *n* = 8). Detergent-insoluble membranes (DIMs) purified from control and Sq-grown pollen tubes showed differences in electrophoretic profile (see black arrows) (DIMs, silver-stained SDS-PAGE; 5 µg of protein in each lane). Western blot analysis of control and Sq-grown DIMs using a monoclonal anti-α-tubulin antibody revealed a significantly lower amount of tubulin associated with Sq-DIMs than control DIMs ((**b**) Student’s *t*-test; *n* = 4). Capital letters in the histograms indicate whether or not there are significant differences.

To check whether treatment with Sq altered the amount of tubulin in pollen tubes, pollen tube crude extracts underwent Western blotting using anti-α-tubulin monoclonal antibody (Figure 2A).

Quantitative analysis of Western blots from five experiments revealed that Sq did not change the total amount of tubulin in Sq-treated pollen tubes with respect to the control (Figure 2A(a)).

To investigate whether the sterol content influenced the interaction of tubulin/MTs with cell membranes, we performed cell fractionation to separate microsomes (P2) from soluble proteins (S2) in control and Sq-treated pollen tubes. The electrophoretic profiles of P2 and S2 obtained from six independent fractionation experiments did not show any difference between control and Sq-treated pollen tube fractions (Figure 2B, gel P2-S2), suggesting that incubation for 3 h with 1 µM Sq did not dramatically affect protein partitioning. In addition, quantitative analysis of Western blots using the anti-α-tubulin monoclonal antibody did not show modifications in tubulin partitioning between the fractions (Figure 2B(a)).

To study whether 1 µM Sq affected the integrity of lipid rafts or the interaction of tubulin/MTs with these specific microdomains, DIMs were purified from tobacco pollen tubes grown with or without Sq. In control and Sq-Optiprep gradients, a floating band of DIMs was observed at the interface between 15% and 30% Optiprep; however, sterol reduction altered the pattern of floating DIMs, since in control gradients, DIMs appeared as thin corpuscles (Figure S2A, control), whereas the floating band obtained from Sq-treated pollen tubes appeared as filamentous/lamellar material (Figure S2A, squalestatin), suggesting that sterol depletion determines structural changes in DIMs. The electrophoretic profiles of fractions derived from the Optiprep gradients showed DIMs in fractions 5–7 in both cases (Figure S2B, brackets; compare control and squalestatin in Optiprep gradients; see also [23]).

Conversely, changes in the electrophoretic profile between control and Sq samples were observed in other regions of the Optiprep gradients (Figure S2B). Changes in the distribution of polypeptides along the Sq gradient, with respect to the control, suggest that sterol depletion could generally alter the sensitivity of pollen tube membranes to detergent, leading to changes in membrane-associated polypeptides and to an altered polypeptide distribution along the Optiprep gradient. On the other hand, although DIMs floated as a single band at the interface between 15% and 30% in the Optiprep density gradient of the control and Sq-treated samples, changes in the patterns of floating material and polypeptides suggested alteration of DIMs due to Sq.

To clarify to what extent the decrease in sterols altered the integrity of DIMs, control and Sq-DIM polypeptides were separated by SDS-PAGE and their electrophoretic profiles were compared (Figure 2B, gel control and Sq-DIMs). The amount of four polypeptides with a molecular mass between 80 and 70 kDa also decreased in Sq-DIMs with respect to the control (Figure 2B, black arrows).

While the results of the P2/S2 analysis did not reveal significant differences in P2/S2 tubulin partitioning between control and Sq samples, we cannot exclude that Sq influenced the interaction of tubulin/MTs with DIMs. DIMs isolated from control and Sq-treated pollen tubes were analyzed by Western blot using a monoclonal antibody against α-tubulin. Quantification and statistical analysis showed that the content of tubulin associated with control DIMs was significantly higher than that associated with Sq-DIMs (Figure 2B(b)). These results suggest that the large amount of tubulin in soluble (S2) and microsomal (P2) fractions masked significant differences in the tubulin content in specific membrane domains like lipid rafts.

### 2.3. Squalestatin Induced Changes in Microtubule Distribution Pattern

**Figure 3:**
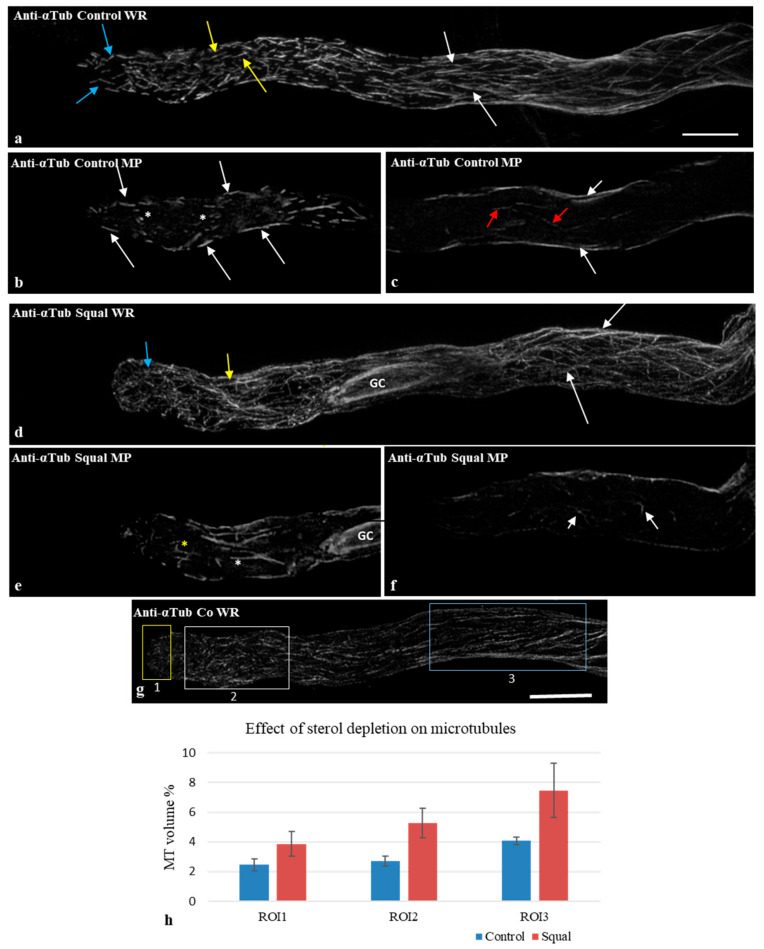
Immunolocalization of microtubules in control and squalestatin-treated pollen tubes. (**a**–**c**) In control pollen tubes, whole reconstructions (WRs) of the cell showed that MTs were arranged in long longitudinal bundles in the distal region ((**a**), white arrows). In the shank and tip regions, randomly oriented short MTs/MT bundles were observed ((**a**), yellow and blue arrows, respectively). Careful observation of medial planes ((**b**,**c**); MPs) revealed that, in control pollen tubes, most MTs localized in the cortex of tip/shank and distal regions ((**b**,**c**), respectively; white arrows), while only very short MT segments or puncta were visible in the tip/shank cytoplasm ((**b**), asterisks). In the distal region, rare cytoplasmic MTs were observed ((**c**), red arrows). (**d**–**f**) In squalestatin-treated pollen tubes, long MT bundles were detected in the distal regions ((**d**), white arrows; WRs). A dense network of MTs was observed in the tip/shank ((**d**), yellow and blue arrows, respectively). In the shank, MTs appeared to be quite long ((**d**), yellow arrow), whereas in the tip region, they were short ((**d**), blue arrow). Microtubules were detected in the generative cell ((**d**), GC). Medial plane observations revealed quite long cytoplasmic MTs in the shank ((**e**), white asterisk; MP) and short MT segments in the tip ((**e**), yellow asterisk). Many cytoplasmic MTs were observed in the distal region (**f**), white arrows). (**g**,**h**). Quantification of MTs in ROI 1-3 (**g**) did not show any differences in the amount of total MTs in the tip, shank and distal regions of control and Sq-treated pollen tubes ((**h**), ROI1–ROI3, two-way ANOVA and post hoc Tukey test, *p* > 0.05). Scale bar = 10 µm.

To explore the relationship between changes in the association between tubulin/MTs and DIMs and the organization of MTs, we studied the MT distribution pattern in control and Sq-treated pollen tubes by indirect immunofluorescence and confocal microscopy. Whole reconstructions (WRs) of control pollen tubes showed that MTs were organized in long bundles in distal regions (up to about 40 µm from the apical PM) (Figure 3a, white arrows).

Short, randomly oriented MTs/MT bundles were detected in the shank (5 µm to 40 µm from the apical PM) (Figure 3a, yellow arrows) and in the apex (5 µm from the apical PM) (Figure 3a, blue arrows). Observation of medial planes revealed short MTs/MT bundles in the cortical regions of the shank and apex (Figure 3b, white arrows), but only rare MT fragments or puncta in the cytoplasmic regions of the shank and tip, respectively (Figure 3b, white asterisks). In the distal regions, observation of medial planes revealed the most MTs in the cortex, and only some short MTs in the cytoplasm (Figure 3c, white arrows).

In pollen tubes grown for 3 h with Sq, MTs organized as long bundles in the distal regions as in control pollen tubes (Figure 3d, WR, white arrows). Microtubule structures in the shank and apical regions appeared thinner than in controls (Figure 3d, yellow and blue arrows, respectively), suggesting the presence of MT bundles with smaller diameters or single MTs (see below). Moreover, in contrast to controls, cytoplasmic MTs/MT bundles were observed in most Sq-treated pollen tubes (Figure 3e, yellow and white asterisks). In particular, cytoplasmic MTs in the shank sometimes crossed or branched from each other (Figure 3e, white asterisks). Cytoplasmic MTs in the apex appeared shorter than in the shank and likewise showed branching or crossing (Figure 3e, yellow asterisk). In pollen tubes treated with Sq, the observation of medial planes in distal regions revealed that, like in the controls, most MTs were located in the cortex (Figure 3f, P). Only a few MTs were observed in the cytoplasm (Figure 3f, white arrows).

To analyze the differences in the content of MTs in different regions of control and Sq-treated pollen tubes, three regions of interest (ROIs) were considered for analysis in the tip, shank and distal regions (yellow, white and blue ROIs, indicated as 1, 2 and 3, respectively; Figure 3g). The percentage volume occupied by MTs in each ROI was determined using Volocity^®^ software (PerkinElmer Inc., Shelton CT, USA). Statistical analysis using two-way ANOVA showed that the percentage volume occupied by total MTs in ROI1-3 was not significantly different in control and Sq-treated pollen tubes (Figure 3h). Moreover, the histogram shows an increasing trend of MT content in all ROIs of Sq samples compared to the control.

Observations of medial planes suggested a greater number of cytoplasmic MTs in the shank of Sq-treated pollen tubes than in controls. Mean fluorescence intensity in the central stacks of the shank (Figure S3, yellow ROI) showed significantly higher mean values of fluorescence in Sq-treated than control pollen tubes (Figure S3(a–c)), confirming a higher number of cytoplasmic MTs in the shank of Sq-treated pollen tubes.

### 2.4. Sterol Depletion Influences the Bundling Activity of Cortical Microtubules in the Apical and Subapical Regions of Pollen Tubes

Immunofluorescence observations suggested that microtubular structures could have smaller width in Sq-treated pollen tubes than in control cells (Figure 3), suggesting that membrane modification affects the ability of MTs to form bundles. To investigate in more detail the bundling ability of cortical MTs, single-molecule localization microscopy (SMLM) experiments were performed in control and sterol-depleted pollen tubes. Cortical MTs were visualized in the apical and subapical regions by indirect immunofluorescence microscopy, as described in the Materials and Methods section.

**Figure 4:**
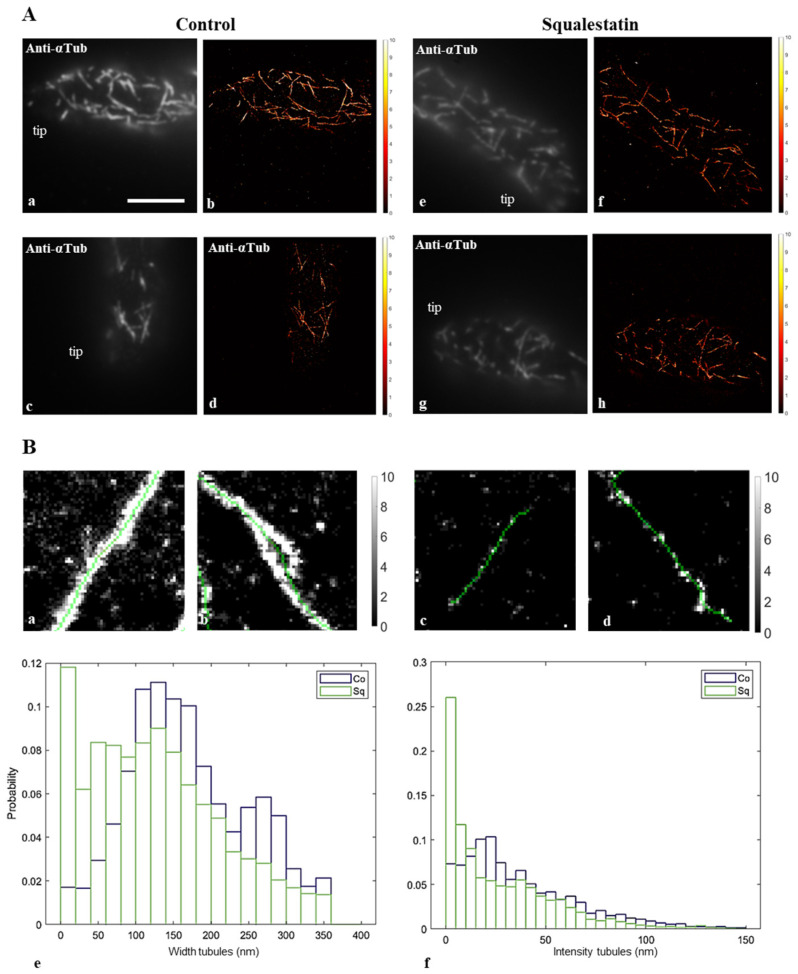
Super-resolution microscopy. (**A**) Cortical microtubule structures (here referred as tubules) observed by super-resolution microscopy in the apical–subapical regions of control pollen tubes (**a**–**d**) appeared thicker than those observed in the same regions of Sq-treated pollen tubes (**e**–**h**). (**B**) Width, fluorescence intensity ((**a**,**b**) control; (**c**,**d**) Sq) and histograms (**e**,**f**) of skeletonized tubules revealed that tubules were significantly thinner in Sq-treated pollen tubes than in controls ((**e**), Kruskal–Wallis test, *p* < 0.0001). Accordingly, the fluorescence intensity of control tubules was higher than that of Sq tubules ((**f**), Kruskal–Wallis test, *p* < 0.0001). Scale bar = 10 µm.

Images of control and Sq-treated pollen tubes from three independent experiments (Figure 4A(a–d) and Figure 4A(e–h), respectively) allowed us to analyze 1863 and 4921 microtubule structures in the apex and subapex (Figure 4B, indicated as tubules).

Tubules detected in control cells were considerably thicker (Figure 4B(a,b)) than those in sterol-depleted cells (Figure 4B(c,d)). The distribution of tubule widths showed clearly that pollen tubes with depleted sterols and altered DIMs had significantly more single MTs (25 nm width) and a higher probability of MT bundles of reduced width than controls (Figure 4B(e)). Consistently, analysis of the distribution of tubule fluorescence intensity revealed significantly higher fluorescence in control than in Sq-treated tubules, corroborating the presence of thicker MT bundles in controls than in Sq-treated pollen tubes (Figure 4B(f)).

These findings suggest that changes in membrane lipid composition, particularly alteration of Lo domains, mostly localized in the apical and subapical PM [23], affect the bundling ability of cortical MTs.

### 2.5. Oryzalin Washout Experiments

Immunofluorescence experiments revealed a higher content of MTs in the shank of Sq-treated pollen tubes than in controls. This difference could depend on changes in microtubule dynamics and/or on altered nucleation activity. To investigate the latter hypothesis, we performed MT recovery experiments after depolymerization in control and sterol-depleted cells.

**Figure 5:**
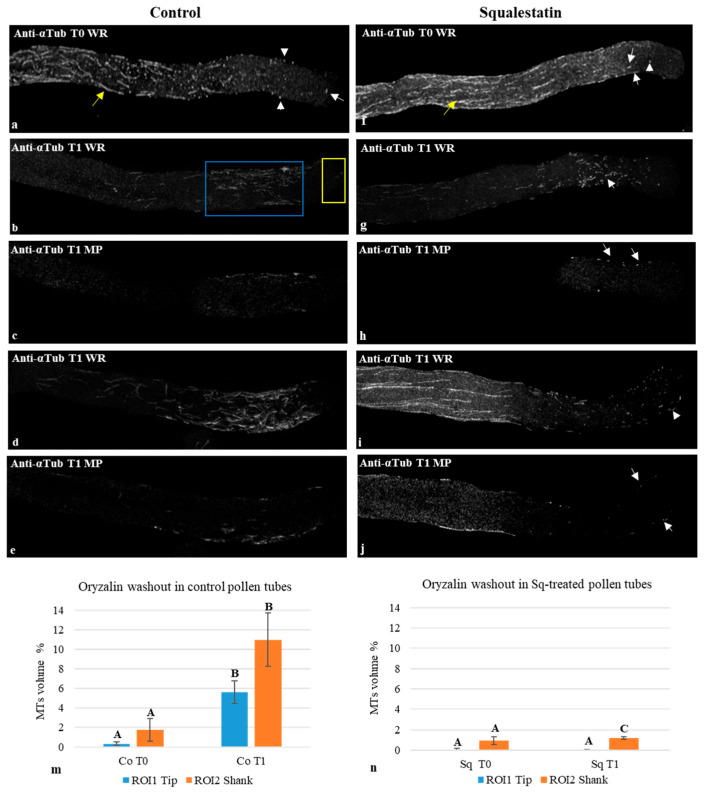
Oryzalin washout. Oryzalin-depolymerized MTs in control and Sq-treated vegetative cells (**a**,**f**). Quantitative analysis at T0 showed that the amount of residual MTs after the oryzalin treatment was not significantly different in the tip ((**b**), yellow ROI) and shank ((**b**), blue ROI) in control and Sq-treated pollen tubes ((**m**,**n**); T0; two-way ANOVA and post hoc Tukey test, *p* > 0.05; *n* ≥ 22). Five minutes after oryzalin washout, MTs were observed more frequently in the shank than in the tip of whole reconstructed control pollen tubes ((**b**,**d**); T1 WR); however, quantitative analysis did not show any significant difference in the amount of MTs recovered from the tip and shank ((**m**), Co T1; two-way ANOVA and post hoc Tukey test, *p* > 0.05; *n* ≥ 31). Furthermore, observation of medial planes showed that MT recovery at T1 occurred in the cortex rather than in the cytoplasmic regions of control pollen tubes ((**c**,**e**); T1 MP). Quantitative analysis showed that MT recovery was significantly faster in control samples compared to squalestatin1 (T1 (**g**–**j**); Two-way ANOVA and post hoc Tukey test, *p* < 0.0001). Scale bar = 10 µm. Capital letters in the histograms indicate whether or not there are significant differences.

Control and Sq-treated pollen tubes were incubated for 5 min with 0.5 µM oryzalin, a strong MT-depolymerizing drug, and aliquots of both samples were processed for indirect immunofluorescence (Figure 5a,f; Co/Sq T0). Oryzalin was immediately washed out and samples were incubated with oryzalin-free medium for a further 5 min to allow for MT recovery (Figure 5b–j, Co/Sq T1).

Oryzalin dramatically affected MT polymerization in the tip and shank of control and Sq-treated pollen tubes after 5 min of incubation (Figure 5a,f; T0). Most cells showed very short MT fragments or puncta in the tip and shank (Figure 5a,f; white arrows and arrowheads, respectively); only a few pollen tubes did not show any MT staining in the apex or shank. On the other hand, in the distal region of pollen tubes grown with or without Sq, microtubules sometimes persisted, albeit fragmented (Figure 5a,f; yellow arrows). These observations suggest that MT bundles were more stable in these areas and therefore less susceptible to the depolymerizing effect of oryzalin. We therefore focused our attention on MT recovery in the apex and shank. Quantification analysis showed that the percentage volume occupied by MTs at T0 in the tip and shank (Figure 5b, yellow and blue ROIs, respectively) was not significantly different in control and Sq-grown pollen tubes (Figure 5m,n).

After oryzalin washout, i.e., at T1, different degrees of MT recovery were observed in the tip and shank of control and squalestatin pollen tubes (Figure 5b,d,m,n). In Sq-grown pollen tubes at T1 (Figure 5g–j,n), only short MT fragments were observed in the shank and sometimes in the tip (Figure 5g,i, WR; white arrow and arrowhead, respectively). Comparison of MT recovery in control and Sq-treated pollen tubes revealed that MT nucleation/regrowth in control cells was significantly more efficient than in Sq cells (Figure 5m,n). Observation of medial planes of control pollen tubes showed short cytoplasmic MTs (Figure 5c,e) whereas in Sq-treated pollen tubes, only tubulin puncta were visible in the cytoplasmic region (Figure 5h,j MP white arrows; Figure S4a,b, yellow arrows).

To analyze in more detail the recovery of cytoplasmic MTs in control and Sq-treated pollen tubes, fluorescence intensity was measured in the medial planes of the shank and tip at T1 (Figure S4a, yellow and blue ROIs, respectively). The recovery of cytoplasmic MTs in the shank was faster than in the tip in both control and Sq-treated pollen tubes (Figure S4c). In terms of tip/shank fluorescence ratio, the recovery of cytoplasmic MTs was more efficient in control than in Sq-treated cells (Figure S4d).

Overall, these data support the hypothesis that alteration of lipid rafts affects the activity of the molecular machinery controlling the nucleation/regrowth of cortical as well as cytoplasmic MTs.

### 2.6. Post-Translational Modification of Tubulins

The increasing trend in the total MT content in Sq-treated pollen tubes and the significant increase in cytoplasmic MTs in the shank may be related to a decrease in MT dynamics. Long-lived MTs are ideal substrates for tubulin post-translational modifications, such as tubulin glutamylation, detyrosination and acetylation, which, in turn, further stabilize tubulin polymers [62,76].

**Figure 6:**
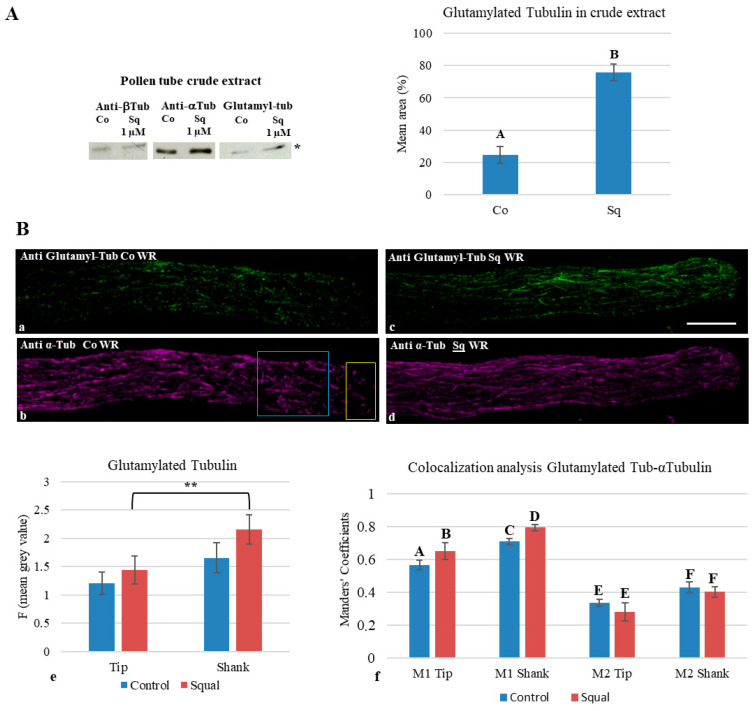
Glutamylated tubulin. (**A**) SDS-PAGE did not show differences in the polypeptide profile of control and Sq-treated pollen tubes. Western blot analysis revealed similar levels of total α- and β-tubulin in the two samples. On the contrary, the amount of glutamylated α-tubulin increased significantly in crude extracts of Sq-grown pollen tubes with respect to control (Western and histogram; Student’s *t*-test, *p*< 0.001; *n* = 6; The identified band is indicated by the asterisk). (**B**) Immunofluorescence microscopy showed glutamylated tubulin in the apical, subapical and distal regions of tobacco pollen tubes of control (**a**,**b**) and Sq-treated cells (**c**,**d**). Analysis of anti-glutamylated tubulin fluorescence intensity (ImageJ, multi-measure option on z-stacks) revealed that glutamylated tubulin in the tip and shank was similar in control and Sq-treated cells (yellow and blue ROIs, (**e**); two-way ANOVA and post hoc Tukey test, *p* > 0.05; *n* ≥ 21). On the contrary, a significant difference was observed between the tip and shank in Sq samples (yellow and blue ROIs, (**e**); two-way ANOVA and post hoc Tukey test, ** *p* = 0.003535; *n* ≥ 21). Colocalization analysis, considering Manders coefficient M2, showed that the fraction of glutamylated MTs did not change between control and Sq-treated pollen tubes ((**f**); two-way ANOVA and post hoc Tukey test, *p* > 0.05), whereas Manders coefficient M1 increased significantly in Sq-treated pollen tubes with respect to control ((**f**); two-way ANOVA and post hoc Tukey test, *p* = 0.013). Scale bar = 10 µm. Capital letters in the histograms indicate whether or not there are significant differences.

The glutamylation, tyrosination and acetylation status of tubulin was investigated by Western blot analysis using anti-glutamylated, anti-tyrosinated and anti-acetylated tubulin monoclonal antibodies on crude extracts of pollen tubes, grown with or without Sq. As already reported in Figure 2, no difference in the electrophoretic pattern (Figure 2A, SDS-PAGE) or in the total tubulin of crude extracts from control and Sq-treated samples was observed (Figure 2A, Western blotting of anti-αtub; a). On the contrary, the amount of glutamylated α-tubulin (Figure 6; compare the migrations of glutamylated tubulin and α/β-tubulins) significantly increased compared to the control in crude extracts of pollen tubes incubated with Sq (Figure 6A; Western blotting of anti-glutamyl tubulin and histogram).

To observe the distribution of glutamylated tubulin on MTs in different regions of pollen tubes, double immunofluorescence experiments were performed on control and Sq-treated pollen tubes using the polyclonal antibody anti-α-tubulin and the monoclonal antibody anti-glutamylated tubulin. The fluorescence intensity of glutamylated tubulin was measured in the tip and shank of both samples (Figure 6B(a–d); yellow and blue ROIs, respectively). Statistical analysis showed a significant increase in glutamylated tubulin in the shank of Sq-treated samples compared to the tip (Figure 6B(e)). Although an increasing trend of tubulin glutamylation was observed between the control and Sq-treated samples, this difference was not significant (Figure 6B(e)).

To test whether this increase in glutamylated tubulin also reflected an increase in the ratio of glutamylated MTs to total MTs in the shank of Sq-treated samples compared to the tip, we performed 3D colocalization analysis (BiopJACoP, ImageJ software), taking Manders coefficients M1 (anti-glutamylated tubulin) and M2 (anti-αtubulin) into account. Manders coefficient M1 increased significantly in Sq-treated pollen tubes compared to the control (Figure 6B(f), yellow and blue ROIs for tip and shank, respectively), indicating a greater amount of glutamylated tubulin in MTs. M2 values showed no difference between control and Sq-treated pollen tubes (Figure 6B(a–d,f)).

**Figure 7:**
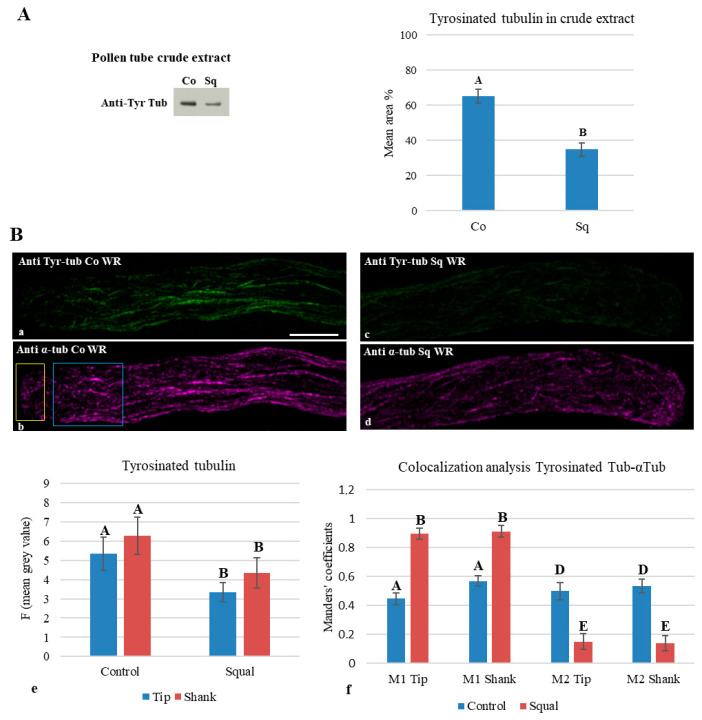
Tyrosinated tubulin. (**A**) The amount of tyrosinated α-tubulin decreased significantly in crude extracts of Sq-grown pollen tubes with respect to controls (Western blot and histogram; Student’s *t*-test, *p* < 0.05; *n* = 4). (**B**) Immunofluorescence microscopy showed tyrosinated tubulin in the apical, subapical and distal regions of control pollen tubes (**a**,**b**). In Sq-treated cells (**c**,**d**), the staining of tyrosinated tubulin decreased along the pollen tubes. In particular, anti-tyrosinated tubulin fluorescence intensity (ImageJ, multi-measure option on z-stacks) revealed that tyrosinated tubulin was significantly lower in the tip and shank (yellow and blue ROIs, respectively) of Sq-treated cells than controls ((**e**), histogram; two-way ANOVA and post hoc Tukey test, *p* = 0.01182 and *p* = 0.01564 for tip and shank, respectively; *n* ≥ 36). Colocalization analysis, considering the Manders coefficient M2, showed that the fraction of tyrosinated MTs decreased significantly in the tip and shank of Sq-treated pollen tubes with respect to controls ((**f**); two-way ANOVA and post hoc Tukey test, *p* < 0.0001), whereas Manders coefficient M1 increased significantly with respect to control ((**f**); two-way ANOVA and post hoc Tukey test, *p* < 0.0001). Scale bar = 10 µm. Capital letters in the histograms indicate whether or not there are significant differences.

Western blot experiments on crude extracts from control and Sq-treated pollen tubes using anti-tyrosinated tubulin antibodies showed that tyrosinated tubulin significantly decreased in cells grown with Sq (Figure 7A, histogram).

Double immunofluorescence experiments were carried out using rabbit anti-α-tubulin polyclonal antibody and anti-tyrosinated tubulin monoclonal antibody. Quantification of mean fluorescence intensity in the tip and shank of both samples (Figure 7B(a–d); yellow and blue ROIs, respectively) confirmed that tyrosinated tubulin decreased significantly in the tip and shank of pollen tubes grown with Sq compared to control (Figure 7B(e)).

To investigate whether the decrease in tyrosinated tubulin also reflected changes in the ratio of tyrosinated MTs to total MTs, we performed 3D colocalization analysis with Manders coefficients M1 (anti- tyrosinated tubulin) and M2 (anti-αtubulin) (plug in BiopJACoP, ImageJ software; Figure 7B(a–d); yellow and blue ROIs for tip and shank, respectively). Although tyrosinated tubulin decreased in Sq-treated pollen tubes, a significantly higher fraction of the remaining tyrosinated tubulin colocalized with tip and shank MTs (Figure 7f; compare M1 values in Co and Sq samples). Moreover, statistical analysis of M2 values showed a significant difference in tyrosinated tubulin between control and Sq-treated pollen tubes (Figure 7B(f)), suggesting that the ratio of tyrosinated to total MTs decreased, and that sterol depletion indeed promotes MT stability by improving tubulin detyrosination.

Western blot analysis and immunofluorescence using anti-acetylated tubulin antibody did not reveal the presence of acetylated tubulin in control and Sq-treated samples.

## 3. Discussion

Various reports show actin and tubulin in DIMs purified from animal and somatic plant cells [20,21,77,78,79]. The interaction between DIMs and the cortical cytoskeleton contributes to lipid raft clustering and cytoskeleton dynamics [80,81,82,83,84,85].

A previous study showed an association of tubulin and actin with DIMs purified from tobacco pollen tubes [23], and that the alteration of lipid rafts affected the morphology and dynamics of the actin fringe [70]. Here, we report the effects of sterol depletion on the organization of MTs in the pollen tubes of *Nicotiana tabacum*. Inhibition of sterol biosynthesis affected the integrity of DIMs, altering their polypeptide profile and reducing the amount of DIM-associated tubulin. The increase in cytoplasmic MTs in the shank and the alteration of cortical MT bundling capacity in the apical and subapical regions of Sq-treated pollen tubes supported the concept that lipid rafts are involved in mediating the interaction of cortical MTs with the PM, and in regulating MT nucleation/regrowth and stability.

### 3.1. Inhibition of Sterol Biosynthesis Delays Pollen Germination

In different cell types, sterols are necessary for the assembly of lipid rafts and treatments affecting sterol biosynthesis influence the integrity of DIMs [67,86]. In a previous study, we showed that the treatment of tobacco pollen tubes with high concentrations of methyl-β-cyclodestrin (MBCD), which removes sterols from the PM, altered DIMs and affected the distribution of Lo domains in the tubes [23]. Moreover, in fractionation experiments, 16 mM MBCD influenced polypeptide distribution between the microsomal and soluble fractions [23], suggesting dramatic effects on pollen tube membranes. Here, treatment of pollen tubes with Sq allowed for finer modulation of the sterol biosynthetic pathway without dramatically affecting the polypeptide profiles of the soluble and microsomal fractions (see below). In treated pollen tubes, squalestatin efficiently lowered total sterols, particularly cycloeucalenol, and delayed pollen germination, suggesting that the integrity of lipid raft microdomains could play a role in pollen tube emission. In rice, Han et al. [36] reported that sterol-enriched microdomains coincided with pollen bulges, further supporting the direct involvement of sterols in establishing the tube emission in mature pollen.

Observations on the organization of MTs during the germination process are rare. Immunofluorescence studies have shown an absence of MTs in the vegetative cell of non-hydrated pollen of *Pyrus communis*. Microtubules only appeared in the vegetative cell after hydration, where they formed a collar of short MTs at the base of emerging pollen tubes. The authors ruled out a direct role of MTs in the germination process, suggesting their indirect role in support of AF dynamics and organization [87].

It would be interesting to investigate the effect of sterol depletion on MT/actin organization in the early stages of pollen germination. We currently know that Sq has an effect on sterol biosynthesis after 2 h of incubation [70], while sterol pattern analysis at shorter times has not been carried out; these points could be a topic of future research.

### 3.2. Sterol Depletion Affects the Interaction of Tubulin with Pollen Tube Detergent-Insoluble Membranes

Cell fractionation experiments in control and Sq-grown pollen tubes revealed that the electrophoretic profile of soluble (S2) and microsomal fractions (P2) derived from control and Sq-treated pollen tubes did not show differences with SDS-PAGE, suggesting that 1 µM Sq did not dramatically alter the interaction of polypeptides with cell membranes.

On the other hand, DIM purification experiments from pollen tubes grown with or without Sq showed differences in the polypeptide profiles of fractions derived from control or Sq-Optiprep gradients, as well as from Sq-DIMs with respect to control DIMs, supporting the idea that sterol depletion affected the integrity of lipid raft microdomains. A previous study on the biochemical characterization of DIMs purified from tobacco pollen tubes revealed polypeptides belonging to several protein classes, such as cytoskeletal proteins and proteins involved in membrane trafficking and lipid metabolism [23]. In the present study, we did not identify polypeptides that differed in quantity between Sq-DIMS and controls, and were therefore unable to postulate that one or more of these pathways were affected by sterol depletion. Further experiments using MALDI TOF/MS are necessary to identify altered polypeptides and will be performed in a future study. Here, we concentrated on the effect of sterol reduction on the relationship between tubulin/MTs and DIMs.

Experiments with the Western blotting of soluble and microsomal fractions, using an antibody against α-tubulin and quantitative analysis, showed that tubulin repartitioned similarly between P2 and S2 in control and Sq-treated samples. On the other hand, Sq-DIMs showed a significantly lower amount of tubulin than controls. As the total tubulin content was not different in control and Sq-treated crude extracts, we postulated a redistribution of tubulin between Lo and Ld domains in pollen tubes with altered lipid rafts.

Although the function of membrane-associated tubulin has not been investigated in pollen tubes, studies of membrane trafficking have revealed that MTs interact with endosomes/vacuoles in *Nicotiana sylvestris* and *Nicotiana tabacum* pollen tubes, regulating vacuole positioning, the trafficking of endosomes and their fusion with vacuoles [40,44]. Moreover, MTs are known to participate in the biogenesis and function of a trans-Golgi network subpopulation and in endoplasmic reticulum remodeling in somatic cells of *Arabidopsis thaliana* [88,89,90]. The emerging functions of MTs in plant membrane trafficking and the connections of cortical MTs with the PM [63,64] imply MT–membrane interactions, so it is not surprising that tubulin partitions between P2 and S2 fractions on homogenization. In particular, the association of tubulin/MT fragments with DIMs in tobacco pollen tubes, despite the presence of Triton X100, indicates that tubulin could be intimately linked to lipid rafts. We can imagine two different scenarios to explain the presence of tubulin in DIMs: single tubulin dimers could interact with lipids rafts, the latter constituting a sort of tubulin storage reserve; alternatively, or additionally, tubulin in DIMs could be derived from interaction of cortical MTs with lipid rafts. This interaction could be direct or mediated by still unidentified MAPs.

Sequence analysis of tubulins showed a few short hydrophobic domains in the dimers, insufficient to justify transmembrane penetration [91]; however, if tubulin cannot penetrate the bilayer, it may be membrane-bound by lipid modifications. Palmitoylation of α- and β-tubulin has been reported prior to the insertion of tubulin in the PM of animal cells [92,93,94]. Membrane solubilization experiments with NP-40 have shown that part of palmitoylated tubulin associates with membrane domains resistant to the action of detergent in the neuronal PM [93,95]. These findings suggest that post-translational lipid modification of tubulin might be responsible for anchoring single dimers and/or cortical MTs to the PM. In any case, direct interaction between palmitoylated tubulin and lipid rafts might not be the only type of interaction of cortical MTs with the PM, since MAPs could also be necessary to mediate some of these connections. In the pollen tubes of tobacco, two polypeptides with molecular masses of 161 kDa and 90 kDa have been identified as putative MAPs, linking cortical MTs to the PM [64]. However, their solubilization by zwitterionic detergents makes it unlikely that these polypeptides link cortical MTs to lipid rafts; rather, they could be involved in the interaction of cortical MTs with Ld domains of the PM [64]. In any case, we cannot exclude that sterol depletion in Ld domains influenced the interaction of MTs with the PM.

### 3.3. Sterols Are Involved in Microtubule Organization

Altered tubulin content in lipid rafts of Sq-treated pollen tubes led us to postulate that sterol depletion affected the organization of cortical MTs. In control pollen tubes, MTs organized in longitudinal bundles in the distal region of the tube, while in the shank and tip, short randomly oriented MTs were observed. Detailed observation of their distribution revealed that MTs were generally distributed in the cortex of vegetative cells and only rarely in the cytoplasm [39,40].

In the present study, immunofluorescence experiments and quantitative analysis showed a significant increase in cytoplasmic MTs in the shank of Sq-grown pollen tubes with respect to controls, although the total MT content was not significantly higher. These data suggest that the large amount of total MTs could mask the slightest variation in cytoplasmic MTs.

The multiple functions of MTs in plant cell morphogenesis and mitosis require rapid transition between an elongation phase and a shortening phase, a process defined as dynamic instability [47]. The increase in cytoplasmic MTs in the shank of Sq-treated pollen tubes could be derived from changes in the ratio of soluble tubulin to MTs, resulting in MT elongation rather than shortening. Sterol-rich domains could possibly function as storage sites for tubulin dimers and the alteration of these domains could release soluble tubulin, altering the tubulin monomer/MT ratio, thereby favoring the elongation of pre-existing MTs and/or the growth of new MTs during the severing–rescue nucleation process [96]. A recent study showed that CLASP (CLIP-associating protein) is involved in stabilizing the severed ends of katanin-generated MT fragments, thus promoting MT growth in Arabidopsis somatic cells [97]. Lindeboom et al. [97] hypothesized that CLASP could favor the rescue of MTs; CLASP could help create cell microenvironments at severing sites, where high concentrations of tubulin promote the growth rather than shrinkage of MTs [47]. The function of CLASP has not been characterized in pollen tubes; however, a fraction of new MTs in the subapical region of pollen tubes could be derived from the severing of pre-existing MTs, and the release of tubulin dimers from altered lipid rafts may enhance the action of CLASP, leading to increased numbers of MTs in the shank.

An alternative hypothesis to explain the increase in cytoplasmic MTs in the shank of Sq-treated pollen tubes could consider that the alteration of lipid rafts due to sterol depletion [23,67,86] may affect interactions between cortical MTs and the PM, enabling cortical MTs to explore the inner part of the cytoplasm. The observation of branched cytoplasmic MTs suggests that cortex-released MTs could also promote branched MT nucleation and/or severing–rescue nucleation processes, leading to a further increase in MTs in this district [98,99].

The hypothesis that cytoplasmic MTs are derived from cortical MT detachment might mean that lipid rafts could offer some anchoring points for MTs with the PM, as observed in neurons [79,95,100,101].

We also have to consider that dynamic instability is not a stochastic process in eukaryotic cells and that a number of MAPs modulate the nucleation and growth of MTs [102]. In pollen tubes, data on the presence of MAPs almost exclusively concern putative kinesin-like polypeptides involved in membrane trafficking [103,104,105]. We can therefore only make suppositions to explain the role of hypothetical MAPs on the instability of MTs. Interactor of constitutive active ROPS (ICRs), which seemed to be involved in root hair growth and MT elongation by mediating ROP signaling pathways, were recently identified in somatic and floral organs [106]. In the pollen tubes of tobacco, the localization of ROP1 in regions with Lo domains and evidence that activation of ROP signaling pathways requires intact lipid rafts [15,36] could be aspects worth investigating for insights into possible relationships between ROP/ICR signaling pathways and MT dynamic instability in pollen tubes.

### 3.4. Sterol Depletion Affected the Bundling Capacity of Cortical MTs

Our single-molecule localization microscopy results indicated that the alteration of the PM lipid composition affected the bundling activity of cortical MTs in apical and subapical regions; thicker MT bundles were detected in control pollen tubes than in Sq-treated cells, which, in turn, showed a greater number of single MTs.

In general, the balance between single MTs and MT bundles might involve the equilibrium between nucleation, stabilization and catastrophe. Bratman and Chang [107] put forward intriguing hypotheses concerning the mechanisms regulating the number of MTs in a bundle. These hypotheses envisaged the possibility that MTOCs sense the number of MTs in the bundles, adding or removing single MTs when necessary. On the other hand, MTOCs might be activated stochastically in concert with the tubulin concentration, thus modulating the number of MTs.

The organization of cortical MTs plays a crucial role in plant cell morphogenesis. A number of studies have shown that the nucleation and bundling activity of cortical MTs are closely interconnected, as MT-dependent MT nucleation helps maintain cortical MT bundles [108].

In plant somatic cells, members of the MAP-65 protein family have been identified as MT-bundling proteins [109,110]. No studies on members of the MAP65 protein family have been conducted in pollen tubes and no other MT-crosslinking proteins have yet been characterized in pollen tubes. However, among proteins favoring MT bundling by regulating PM-MT interactions, there are members of the CLASP family. Besides playing a role in stabilizing MT seeds and promoting MT elongation during the severing–rescue nucleation process (see below), members of the CLASP family are also involved in maintaining cortical MT bundles in somatic plant cells [97,111,112]. In CLASP mutants, MT bundles appear thinner than in control cells, reflecting fewer MTs in each bundle [111,113]. It is believed that members of the CLASP family bind to plus ends of nascent MTs and, at the same time, mediate interaction of these MTs with the PM, thus favoring MT encounters in the cortex of somatic cells and MT bundling [60,111,113].

No studies on the function of CLASP have been reported in pollen tubes; however, CLASP-like or other as yet uncharacterized proteins could be involved in anchoring the plus end of nascent MTs to the cortex, stabilizing new MTs and favoring bundled rather than single MTs. Moreover, in CLASP mutants, MTs show a higher frequency of detachments from the PM than in control cells [98,111,113]. In pollen tubes incubated with Sq, a smaller amount of sterols might have affected the interaction of CLASP homologues/nascent MTs with the PM in the apical and subapical regions of pollen tubes, where Lo domains are concentrated [23], thus affecting their incorporation into cortical bundles. Consequently, a proportion of new MTs may have detached from the cortex to explore the inner part of the cytoplasm, themselves becoming platforms for cytoplasmic MT-dependent or severing–rescue MT nucleation of cytoplasmic MTs.

### 3.5. Sterol Depletion Affected MT Nucleation

The results of oryzalin washout experiments showed that, in Sq-treated pollen tubes, MT recovery was significantly slower than in control cells, suggesting that alteration of lipid rafts had a greater effect on the lag phase of MT nucleation/regrowth after oryzalin washout in treated than in control pollen tubes.

Microtubule nucleation modes have yet to be studied in depth in pollen tubes. Use of a heterologous antibody against a mammalian pericentriolar protein has suggested that MTOCs associate with the apical and subapical PM [114], reflecting the localization of Lo domains [23]. Based on these data, we can postulate that, in control cells, most MT nucleation occurs in the cortex of apical and subapical regions. In Sq-treated pollen tubes, where MT recovery was slower than in control pollen tubes, the alteration of sterol content may disturb the interaction of γ-tubulin ring complexes (γTuRCs) with the PM, lengthening the nucleation lag phase and delaying MT nucleation.

In eukaryotes, γTuRC protein aggregates work as initial templates for MT nucleation [115,116,117]. In plant cells, γTuRCs associate with the nuclear envelope surface, the cell cortex and the surface of pre-existing MTs, the latter giving rise to branched nucleation [59,96,99,118,119,120,121]. Interestingly, in mammalian cells, MTs nucleated by γTuRCs at the Golgi apparatus are reported to be stabilized by CLASP proteins [122]. Although CLASP proteins have not been studied in pollen tubes, pollen tube CLASP homologues could possibly stabilize new MT seeds and simultaneously mediate the interaction of new MT plus ends with the PM in order to stabilize and promote MT growth. In this view, modification of the PM lipid profile and, in particular, that of lipid raft microdomains, impaired the interaction of new MT seeds with the PM, thus retarding MT regrowth after oryzalin depolymerization.

### 3.6. Sterol Depletion Affected Post-Translational Modifications of Tubulin

Many studies in animal and plant cells have shown that the expression of specific α- and β-tubulin isotypes and the tubulin post-translational modification program give rise to different MT arrays for specific functions [76,123,124].

Although many studies in animal cells help elucidate the role of post-translational modifications of tubulins, limited information is available for plant cells [53]. Most information has come from immunofluorescence studies using antibodies directed against tubulins modified during the cell cycle; the distribution of glutamylated, tyrosinated/detyrosinated and acetylated tubulins predicts the dynamics of MT arrays. In the pollen tubes of angiosperms, acetylated MTs have only been detected in pollen tubes treated with taxol [41]. Western blot analysis and immunofluorescence experiments using anti-acetylated tubulin were inconclusive in pollen tubes grown in control medium or with squalestatin.

Most α-tubulin genes in different organisms encode a tyrosine at the carboxy end; terminal tyrosine can be removed from MTs, leading to detyrosinated tubulin [125]. On the other hand, tubulin tyrosine ligase catalyzes the reverse reaction by adding a tyrosine to tubulin monomers [126]. This mechanism, together with the tubulin-detyrosinating enzyme’s preference for polymerized MTs, results in detyrosinated tubulin accumulating in stable microtubules [127,128,129]. In mammalian cells, the tyrosinated/detyrosinated tubulin ratio also modulates the interaction of MAPs with MTs [130,131,132,133,134].

Tubulin polyglutamylation occurs preferentially on MTs by the addition of glutamate side chains to α- and β-tubulin carboxy-terminal domains by members of the tubulin tyrosine ligase-like (TTLL) family [135,136]. This process is reversible, as cytosolic carboxypeptidases (CCP) and glutamylases can remove these side chains and modulate their length, respectively [137,138]. Early studies on tubulin glutamylation revealed that the addition of glutamate side chains is associated with long-lived MTs [139,140]. As in the case of tyrosination/detyrosination, the level carboxy-terminal glutamylation of tubulin regulates the interaction of MTs with MAPs [134,141].

In pollen tubes grown with squalestatin, a significant increase in glutamylated α-tubulin with respect to the control was recorded by Western blot analysis. The immunofluorescence and the colocalization analysis showed that the level of glutamylation was also higher in shank than in tip MTs in Sq samples, in line with the greater abundance of cytoplasmic MTs in the shank.

In tobacco pollen tubes, tyrosinated tubulin is found in all MT subpopulations: in randomly oriented MT fragments in the apex and shank, as well as in the long MT bundles in the distal region, suggesting that pollen tube growth requires dynamic MTs [39]. In pollen tubes grown with squalestatin, a significant reduction in tyrosinated tubulin was detected by Western blot analysis and immunofluorescence microscopy in the tip and shank. Double immunofluorescence experiments confirmed that the fraction of tyrosinated MTs dramatically decreases in sterol-depleted pollen tubes. These findings suggest that lipid microdomains could be involved in tyrosination/detyrosination processes, or alternatively, that MT detyrosination/glutamylation could be compensating strategies to stabilize single MTs, found preferentially in pollen tubes low in sterols.

## 4. Conclusions

This paper provides novel insights into the role of lipid rafts microdomains as factors that could regulate the dynamics and organization of MTs in angiosperm male gametophytes. Lipid raft microdomains could therefore represent platforms for creating microenvironments in which protein complexes involved in these processes are concentrated.

In discussing the data, we proposed various scenarios. Though intriguing, these hypotheses need to be verified by identifying MAPs and, particularly, the possible associations and dynamics of γ-tubulin and members of the CLASP protein family with microsomes and lipid rafts.

The study of the relationship between cytoskeletal elements and lipid rafts will provide new insights into the mechanisms underlying sexual reproduction in plants. This knowledge, in addition to being of great cultural value, could be extremely important for specific applications in agriculture, for example, in studying self-incompatibility or temperature aspects associated with global warming.

We would like to point out that, looking ahead, the study of the apical growth mechanisms of male gametophytes in angiosperms could provide new insights for investigating this process from an evolutionary perspective (including the protonemata of mosses).

## 5. Materials and Methods

### 5.1. Germination Assay and Pollen Tube Measurement

Pollen of *Nicotiana tabacum* (L.) was collected from plants grown in the Botanical Garden (Città Studi) of Milan University during summer. Anthers were left to open in a Petri dish for 2 days, completely dehydrated for 12 h in a box containing silica gel and then stored at −20 °C. Pollen grains were hydrated in a humid chamber overnight and cultured in flasks (3 mg/mL) in BK liquid medium [142] supplemented with 12% (*w*/*v*) sucrose at 23 ± 2 °C with or without 0.5 or 1 µM Sq for 3 and 5 h. Although pollen was not tested before freezing, its germinability was tested during germination in a BK medium, taking into account the amount of pollen grains forming a pollen tube and the length of the pollen tubes.

The length of pollen tubes grown with or without squalestatin was calculated with ImageJ software (National Institutes of Health) [143]. Control and treated pollen tubes were observed after chemical fixation (see below) with a Leica DMRB microscope (Leica N PLAN 10X objective), and images were taken with a Leica MC 170 HD video camera. For the germination assays, at least 10 optical fields of control and Sq-treated samples were acquired, as reported above, in three independent experiments.

### 5.2. Probes and Drugs

Zaragozic acid/squalestatin (Sq) (Sigma Aldrich, St. Louis, MO, USA) was dissolved in ethanol to a concentration of 6.6 mM and then diluted to concentrations of 1 µM in BK medium (see below). Oryzalin (stock solution, 1.38 M) was used at a final concentration of 0.5 µM.

Anti-α tubulin polyclonal antibody was purchased from Agrisera (Vännäs, Sweden). Anti-α- (clone B-5-1-2), anti-β- (clone TUB 2.1), anti-tyrosinated- (clone TUB-1A2), anti-polyglutamylated- (clone B3) and anti-acetylated-tubulin (clone 6-11B-1) monoclonal antibodies were from Sigma (St. Louis, MO, USA). Texas-Red-conjugated anti-rabbit, FITC-conjugated anti-mouse and Alexa Fluor plus 647-conjugated anti-mouse antibodies were obtained from Invitrogen (Waltham, MA, USA). ECL peroxidase-labeled anti-mouse secondary antibody for Western blot assays was provided by EUROCLONE (Pero, Italy).

### 5.3. Pollen Tube Crude Extract

*Nicotiana tabacum* (L.) pollen was germinated in BK medium (3 mg/mL), as reported above, for 3 h with or without Sq 1 µM. Pollen tubes were collected by centrifuging at 2000 rpm for 10 min at 10 °C in a Beckmann JS13.1 rotor, and then rinsed with 10 mL incomplete TNE buffer (50 mM Tris-HCl, pH 7.4; 150 mM NaCl; 5 mM EDTA; 10 µg/mL TAME; 1 mM PMSF) containing 12% sucrose, with or without Sq 1 µM. Samples were then centrifuged at 2000 rpm for 10 min at 10 °C in the same centrifuge. Control and Sq-treated pollen tubes were resuspended in two volumes of cold complete TNE buffer (50 mM Tris-HCl, pH 7.4; 150 mM NaCl; 5 mM EDTA; 1 mM PMSF; 10 µg/mL TAME; 10 µg/mL leupeptin; 10 µg/mL pepstatin A; 4 µM aprotinin; 8 µM antipain) and homogenized on ice in a 2 mL Potter homogenizer (Teflon/glass; 80 stocks). Laemmli Sample Buffer (LSB, see below) without Bromophenol Blue [144] was added to the homogenate. Samples were boiled for 2 min and then centrifuged at 10 °C for 40 min at 15,000 rpm in an ALC A21-C rotor. The resulting supernatants were collected as crude extracts (CE control/Sq). Aliquots of CE were protein assayed using BSA as standard protein [145].

### 5.4. Cell Fractionation

Microsomal and soluble fractions (control and Sq-P2) were prepared by the protocol reported by Moscatelli et al. [23]. Control and Sq-treated pollen tubes were processed as reported in the section describing the procedure for crude extract. The homogenates were centrifuged at 2500 rpm for 4 min at 4 °C in an ALC A21-C rotor. Pellets (P1 Co/Sq) were discarded, and control and Sq post-nuclear supernatants (S1 Co/Sq) were loaded onto 20% sucrose cushions (3 mL) in incomplete TNE buffer and centrifuged at 23,000 rpm for 30 min at 4 °C in the Beckman SW-60 Ti rotor. Control and Sq pellets (microsomes, P2) were resuspended in cold, complete TNE buffer. Aliquots of P2 and supernatants (S2) were protein-assayed using BSA as standard protein [145]. Aliquots of control and Sq-P2 were dried in a Speed Vac SC110 (Savant) and stored at −80 °C for analysis of lipid profile.

### 5.5. HPTLC Analysis of Lipids

For the analysis of sterols, P2 fractions obtained from pollen tubes grown with or without 0.5 or 1 µM Sq for 3 and 5 h were resuspended in 0.5 mL H2O, to which 2 mL chloroform/methanol (2/1, *v*/*v*) was added, and vortexed. After 1 h, the tubes were centrifuged at 3000× *g* for 10 min and the supernatant was evaporated to dryness. The lipid extracts were analyzed by HPTLC or further processed for GC-MS. For HPTLC: lipids were resuspended in chloroform/methanol (2/1, *v*/*v*) and chromatography was performed using hexane/ethyl ether/acetic acid (90/15/2, *v*/*v*). For GC-MS: saponification was carried out by adding 1 mL ethanol and 0.1 mL KOH 11 N to the lipid extracts, as well as 5 μg cholesterol as internal standard, and incubating the tubes at 80 °C for 1 h. Sterols were extracted with 1 mL hexane and then 2 mL H2O was added; tubes were then vortexed and centrifuged at 3000× *g* for 10 min. The supernatant was transferred to another tube and evaporated to dryness under a nitrogen stream before silylation. Sterols were derivatized with 200 μL N,O-bis(trimethylsilyl)trifluoroacetamide with 0.1% trimethylchlorosilane (BSTFA/TMCS) and incubated at 110 °C for 15 min. BSTFA was evaporated under a nitrogen stream and the samples were dissolved in hexane. GC analysis was carried out with an Agilent 7890A Plus GC unit (Agilent, Santa Clara, CA, USA) with a flame ionization detector (Agilent). Silylated sterols were separated on a 30 m HP-5MS column (Agilent) using a temperature gradient of 150 °C; this was increased to 280 °C at 10 °C min^−1^ and held for 10 min, and then decreased to 150 °C at 20 °C min^−1^. Sterols were identified by GC-MS (Agilent) using the same column and temperature gradient.

### 5.6. Preparation of Detergent-Insoluble Membranes (DIMs)

Tobacco pollen (two 400 mg aliquots for control and Sq samples) was cultured for 3 h in BK medium supplemented with 12% sucrose, with or without 1 µM Sq, as reported above. Control and Sq-treated pollen tubes were processed as reported in the section describing the crude extract procedure. Control and Sq-treated pollen tubes were homogenized on ice in two volumes of complete TNE buffer using 5 mL Potter homogenizers (Teflon/glass; 80 stokes). Control and Sq microsomes (P2) were prepared as described in the previous sections (cell fractionation). Control and Sq P2 samples were resuspended in cold complete TNE buffer and ice-cold 10% (*w*/*v*) Triton X 100 was added to an 8:1 detergent/P2 protein mixture (1% final concentration) [23]. After treatment with 1% Triton for 30 min on ice, membranes were mixed with 60% Optiprep (from Sigma, St. Louis, Missouri, USA) to a final concentration of 40% (*v*/*v*), placed at the bottom of a centrifuge tube and overlaid with successive 3 mL steps of 35%, 30% and 15% (*v*/*v*) Optiprep/TNE buffer. Gradients were centrifuged for 19 h at 37,000 rpm in a Beckmann SW41 Ti rotor. DIMs were recovered as opaque bands of floating material at the interface between 15% and 30% Optiprep. Control and Sq gradients were separated into 19 fractions and aliquots of each fraction were denatured for electrophoresis. The same amount of protein was loaded in the two gradients. The same volume of each gradient fraction (25 µL) was loaded on the gel. Silver staining revealed polypeptides. Fractions corresponding to the floating material were pooled, diluted with 4 volumes of TE buffer (50 mM Tris-HCl, pH 7.4; 5 mM EDTA; 1 mM PMSF; 10 mg/mL TAME) and centrifuged at 32,000 rpm for 2 h at 4 °C in a Beckmann SW 60 Ti rotor. Aliquots of control and Sq-DIMs were assayed for protein concentration [145] and stored at −80 °C.

### 5.7. SDS-PAGE and Western Blot

Proteins of crude extracts (15 µg/lane), P2/S2 samples (15 μg/lane), fractions of Optiprep gradients (20 µL fraction/lane) and DIMs (5 µg for control and Sq DIMs/lane) were resolved in denaturing 10% polyacrylamide one-dimensional (1D) gels in a discontinuous buffer system [144]. A MiniVe Vertical Electrophoresis System (GE Healthcare, Chicago, IL, USA) was used for 1D electrophoresis. Polypeptides were revealed by staining the gels with Coomassie brilliant blue R250 or by silver enhancement [146]. Images of gels were acquired using Epson Perfection V750 PRO and Adobe Photoshop software. Western blots were performed according to Towbin et al. [147]. Membranes were blocked in 5% non-fat milk in Tris-buffered saline (TBS) at room temperature. They were probed with primary monoclonal antibodies diluted 1:2000 overnight at room temperature. Secondary anti-mouse antibody was used at a 1:10,000 final dilution.

### 5.8. Indirect Immunofluorescence

Pollen tubes grown in control BK medium with or without 1 μM Sq for 3 h were processed for indirect immunofluorescence. Samples were incubated in fixing solution (3.7% formaldehyde; 12% sucrose; 100 mM PIPES; 5 mM MgSO_4_; 0.05 mM CaCl_2_; pH 6.9). For single immunofluorescence, cells were treated as described in [45]; pollen tubes were incubated with rabbit anti-α-tubulin polyclonal antibody at a concentration of 1:200 for 15 h at 4 °C. A Texas-Red-conjugated anti-rabbit secondary antibody was used at 1:200 final dilutions. Optical sections (0.2 μm) and 3D projections of specimens were obtained with a Leica TCS NT SP8 point scanning confocal microscope and a 63X/1.4 objective. Texas Red fluorescence was excited with the 561 nm line from a white light laser selected with an acousto-optic tunable filter, and collected with a photomultiplier tube having an emission window of 600–700 nm.

### 5.9. Double Immunofluorescence and Colocalization Analysis

For double immunofluorescence, pollen tubes grown in control medium or in medium supplemented with 1 µM Sq were treated as reported in the previous section. Cells were incubated sequentially with primary antibodies: anti-α-tubulin polyclonal antibody (1:200 for 2 h at room temperature) and anti-tyrosinated or anti-glutamylated tubulin monoclonal antibodies (1:200 for 15 h at 4 °C). As secondary antibodies, Texas-Red-conjugated anti-rabbit and FITC-conjugated anti-mouse antibodies were used at a 1:200 final dilution for 2 h at room temperature. Specimens were observed with a Leica TCS NT SP8 confocal microscope, as reported above. A sequential scan to acquire the FITC-conjugated signal was added: 488 nm laser line and 494–550 nm collection emission range.

### 5.10. Oryzalin Washout Experiments

For oryzalin washout experiments, control pollen tubes and pollen tubes treated with 1 µM Sq for 3 h were incubated with oryzalin 0.5 µM for 5 min (Co/Sq T0). Pollen tubes were rinsed with 2 mL of medium with or without Sq and left to recover for 5 min (Co/Sq T1). Cells were fixed at T0 and T1 and processed by indirect immunofluorescence, observing MTs with a Leica TCS NT SP8 confocal microscope and 63X/1.4 objective.

### 5.11. Single-Molecule Localization Microscopy

For single-molecule localization imaging experiments, pollen tubes grown with or without 1 µM Sq were treated as described for indirect immunofluorescence [40], except that anti-α-tubulin monoclonal antibody (clone TUB 2.1) was used as a primary antibody and Alexa Fluor 647-conjugated anti-mouse antibody as a secondary antibody. Single-molecule localization imaging was obtained by direct stochastic optical reconstruction microscopy dSTORM [148] using a GSD microscope (Leica SR GSD, Leica Microsystems, Mannheim, Germany) equipped with a solid-state 642 nm laser, an oil immersion objective lens (HCX PL APO 160x 1.45NA) and an EMCCD camera (Andor iXon Ultra-897, Oxford Instruments, Oxford, UK). All dSTORM experiments were performed with Smart-kit buffer (Abbelight, Cachan, France). We activated real-time localization with the laser in an oblique configuration (Hilo), an integration time of 20 ms and an EMCCD gain set at 300.

### 5.12. Quantification and Statistical Analysis

Values of total sterols after 3 and 5 h germinations were analyzed using the one-way ANOVA test (IBM SPSS Statistics. v.29.0). Tukey’s HSD post hoc test was used to assess the significance of each comparison.

To compare control and Sq-treated pollen tubes after 3 and 5 h of incubation, the pollen tube length of control and Sq-treated samples at different time points was compared using Student’s *t*-test with the Excel program.

For the germination assay, nongerminated pollen grains (U), pollen grains with germination bubbles (GBs), pollen with short pollen tubes (pollen tubes less than twice the grain diameter; STs) and germinated pollen (pollen tubes at least twice the grain diameter; G) were counted after 1 h in BK medium in three independent experiments. The number of U, GB, ST and G pollen grains in control and Sq-treated samples after 1 h in BK medium was compared by the chi-square test (IBM SPSS Statistics. v.29.0).

For MT quantification in control and in Sq-treated samples, acquired z-stacks were deconvoluted (Huygens Professional software, Scientific Volume Imaging, The Netherlands, http://svi.nl). Quantitative analysis of the area covered by MT staining in tip (ROI1), shank (ROI2) and distal (ROI3) regions of pollen tubes were performed by creating a measurement protocol using Volocity software (Volocity^®^ PerkinElmer Inc., Shelton CT, USA). Once images were transferred into Volocity software, 3 ROIs were selected manually for each pollen tube in order to identify the tip (ROI1 5 μm wide), a portion just below the tip (ROI2 20 μm wide) and a portion distant 25 μm from ROI2 (ROI3, 15 μm wide). In each ROI, the total volume of the tube was identified as the “full object”, setting the minimum intensity threshold quite low, while MTs were segmented with a specific threshold on the correct channel.

For the oryzalin washout assay, quantitative analysis of MTs in control and Sq-treated pollen tubes at T0 and T1 was carried out with Volocity software (Volocity^®^, PerkinElmer Inc.) in the tip (ROI1) and shank (ROI2). Images acquired by an SP8 Leica point scanning confocal microscope were first deconvoluted with Huygens Professional version 19.04 software (Scientific Volume Imaging, The Netherlands, http://svi.nl) using the CMLE algorithm, with an SNR of 10 and 40 iterations. Once the data were transferred to Volocity software, two ROIs for each pollen tube were selected manually to identify the tip (ROI1, 5 µm wide) and a portion just below the tip (ROI2, 20 µm wide). In each ROI, the total volume of the tube was identified as the “full object”, setting the minimum intensity threshold quite low, while MTs were segmented with a specific threshold on the correct channel. The results were exported into Excel and analyzed for significant differences.

In both cases, the values of the percentage volume occupied by MTs in each ROI in control and treated cells were processed by statistical analysis. The two-way ANOVA test (IBM SPSS Statistics. v.29.0) was used to evaluate the difference between ROIs in different experimental conditions. Tukey’s HSD post hoc test was used to assess the significance of each comparison. To estimate the difference in cytoplasmic MTs between control and Sq-treated pollen tubes, the mean fluorescence intensity was calculated in the shank cytoplasm of single pollen tubes using ImageJ software (Rasband, W.S., ImageJ, US National Institutes of Health, Bethesda, MD, USA, http://imagej.nih.gov/ij/, 1997–2012) [144]. Five medial sections were considered for each pollen tube (ImageJ, multi-measure option on Z-axis). Values were analyzed by two-way ANOVA (IBM SPSS Statistics. v.29.0); Tukey’s HSD post hoc test was used to assess the significance of each comparison.

Data derived from single-molecule localization microscopy were analyzed using a custom Matlab pipeline. Images were reconstructed from the localization list with a pixel size of 20 nm, corresponding to the resolution of the STORM technique. Tubule segmentation was performed using the Frangi filter [149] to enhance elongated structures, followed by global thresholding. The Matlab implementation of the Frangi filter was obtained from an online repository [150] (Tim Jerman, 2022). The segmented tubule mask was skeletonized and divided into individual branches. Straight (non-bent) elements were selected and rotated to align with the pixel grid. Rotation was applied to the localization list and the STORM image was subsequently reconstructed to ensure correct local orientation. Tubule length and width were measured by counting pixels along the longitudinal and transverse axes, respectively. Statistical differences between distributions were evaluated using the Kruskal–Wallis test.

In double fluorescence–colocalization experiments, the mean fluorescent intensity of tyrosinated and glutamylated tubulin in the apex (ROI1) and shank (ROI2) of single pollen tubes was calculated by ImageJ (multi measure option on Z axis; Rasband, W.S., ImageJ, US National Institutes of Health, Bethesda, Maryland, USA, http://imagej.nih.gov/ij/, 1997–2012) [145]. Values were processed for statistical analysis (Student’s *t*-test) using Excel. Three-dimensional colocalization analysis was conducted with the plug-in BiopJACoP using ImageJ software [151]. The proportion of MTs containing polyglutamylated or tyrosinated α-tubulin was estimated by calculating the Manders coefficients M1 and M2 in the apex and shank regions. Manders coefficients were calculated using the same ROIs in all images. Values were compared using two-way ANOVA; Tukey’s HSD post hoc test was used to assess the significance of each comparison (IBM SPSS Statistics. v.29.0).

For the quantification of Western blotting, bound antibodies were detected with the enhanced chemiluminescence reagent ECL (Thermo Scientific). Western blot images were acquired with a Chemidoc system and Image Lab software (version 5.2 1 Bio-Rad, Hercules, CA, USA). Western blot image quantification was performed with ImageJ software (National Institutes of Health) [145] on the basis of band areas. Values were processed for statistical analysis (Student’s *t*-test) using Excel.

## Data Availability

The results of this study are stored on a laboratory storage device and are available upon request from the corresponding author (Alessandra Moscatelli).

## Supplementary Materials

The following supporting information can be downloaded at: https://www.mdpi.com/article/10.3390/plants14243845/s1, Figure S1: Effect of squalestatin on pollen tube growth.; Figure S2: Optiprep gradients; Figure S3: Effect of squalestatin on cytoplasmic MTs; Figure S4: Recovery of cytoplasmic MTs after oryzalin washout.

## Abbreviations

The following abbreviations are used in this manuscript:

PMP	Plasma membrane
Lo	Liquid-ordered domains
Ld	Liquid-disordered domains
DIMs	Detergent-insoluble membranes
AFs	Actin filaments
MTs	Microtubules
Sq	Squalestatin
Myr	Myriocin
MAPs	Microtubule-binding proteins
WR	Whole reconstruction
MP	Medial plane
GC	Generative cell

## References

[1] Navashin S.G. (1898). Resultate einer revision der befruchtungsvorgange bei *Lilium martagon* und *Fritillaria tenella*. Bull. Acad. Sci. St. Petersburg.

[2] Guignard M.L. (1899). Sur les antherozoides et la double copulation sexuelle chez les vegetaux angiosperms. Rev. Gén. Bot..

[3] Vidali L., McKenna S.T., Hepler P.K. (2001). Actin polymerization is essential for pollen tube growth. Mol. Biol. Cell.

[4] Cardenas L., Lovy-Wheeler A., Wilsen K.L., Hepler P.K. (2005). Actin polymerization promotes the reversal of streaming in the apex of pollen tubes. Cell Motil. Cytoskel..

[5] Cheung A.Y., Wu H.M. (2008). Structural and signalling networks for the polar cell growth machinery in pollen tubes. Ann. Rev. Plant Biol..

[6] Singer S.J., Nicolson G. (1972). The fluid mosaic model of the structure of cell membranes. Science.

[7] Simons K., Ikonen E. (1997). Functional rafts in cell membranes. Nature.

[8] Ohvo-Rekilä H., Ramstedt B., Leppimäki P., Slotte J.P. (2002). Cholesterol interactions with phospholipids in membranes. Prog. Lipid Res..

[9] Silvius J.R. (2003). Role of cholesterol in lipid raft formation: Lessons from lipid model systems. Biochim. Biophys. Acta.

[10] Lingwood D., Simons K. (2010). Lipid rafts as a membrane organization principle. Science.

[11] Jaillais Y., Ott T. (2020). The nanoscale organization of the plasma membrane and its importance in signaling: A proteolipid perspective. Plant Physiol..

[12] Schroeder R., London E., Brown D. (1994). Interactions between saturated acyl chains confer detergent resistance on lipids and glycosylphosphatidylinositol (GPI)-anchored proteins: GPI-anchored proteins in liposomes and cells show similar behavior. Proc. Natl. Acad. Sci. USA.

[13] Brown D.A., London E. (1997). Structure of detergent-resistant membrane domains: Does phase separation occur in biological membranes?. Biochem. Biophys. Res. Commun..

[14] Muñiz M., Zurzolo C. (2014). Sorting of GPI-anchored proteins from yeast to mammals--common pathways at different sites?. J. Cell Sci..

[15] Fratini M., Krishnamoorthy P., Stenzel I., Riechmann M., Matzner M., Bacia K., Heilmann M., Heilmann I. (2021). Plasma membrane nano-organization specifies phosphoinositide effects on Rho-GTPases and actin dynamics in tobacco pollen tubes. Plant Cell.

[16] Ahmed S.N., Brown D.A., London E. (1997). On the origin of sphingolipid/cholesterol-rich detergent-insoluble cell membranes: Physiological concentrations of cholesterol and sphingolipid induce formation of a detergent-insoluble, liquid-ordered lipid phase in model membranes. Biochemistry.

[17] Brown D.A., London E. (2000). Structure and function of sphingolipid- and cholesterol-rich membrane rafts. J. Biol. Chem..

[18] Dietrich C., Bagatolli L.A., Volovyk Z.N., Thompson N.L., Levi M., Jacobson K., Gratton E. (2001). Lipid rafts reconstituted in model membranes. Biophys. J..

[19] London E. (2002). into lipid raft structure and formation from experiments in model membranes. Curr. Opin. Struct. Biol..

[20] Mongrand S., Morel J., Laroche J., Claverol S., Carde J.P., Hartman M.A., Bonneu M., Simon Plas F., Lessire R., Bessoule J.J. (2004). Lipid rafts in higher plant cells: Purification and characterization of Triton X-100-insoluble microdomains from tobacco plasma membrane. J. Biol. Chem..

[21] Borner G.H., Sherrier D.J., Weimar T., Michaelson L.V., Hawkins N.D., Macaskill A., Napier J.A., Beale M.H., Lilley K.S., Dupree P. (2005). Analysis of detergent-resistant membranes in Arabidopsis. Evidence for plasma membrane lipid rafts. Plant Physiol..

[22] Cacas J.L., Furt F., Le Guedard M., Schmitter J.M., Buré C., Gerbeau-Pissot P., Moreau P., Bessoule J.J., Simon-Plas F., Mongrand S. (2012). Lipids of plant membrane rafts. Prog. Lipid Res..

[23] Moscatelli A., Gagliardi A., Maneta-Peyret L., Bini L., Stroppa N., Onelli E., Scali M., Idilli A.I., Moreau P. (2015). Characterization of detergent-insoluble membranes in pollen tubes of *Nicotiana tabacum* (L.). Biol. Open.

[24] Shimmen T. (2007). The sliding theory of cytoplasmic streaming: Fifty years of progress. J. Plant Res..

[25] Cardenas L., Lovy-Wheeler A., Kunkel J.G., Hepler P.K. (2008). Pollen tube oscillatory and intracellular calcium levels are reversibly modulated by actin polymerization. Plant Physiol..

[26] Zhang Y., He J., Lee D., McCormick S. (2010). Interdependence of endomembrane trafficking and actin dynamics during polarized growth of Arabidopsis pollen tubes. Plant Physiol..

[27] Lovy-Wheeler A., Wilsen K.L., Baskin T.I., Hepler P.K. (2005). Enhanced fixation reveals the apical cortical fringe of actin filaments as a consistent feature if the pollen tube. Planta.

[28] Vidali L., Round C.M., Hepler P.K., Bezanilla M. (2009). Lifeact-mEGFP reveals a dynamic apical Factin network in tip growing plant cells. PLoS ONE.

[29] Rounds C.M., Hepler P.K., Winship L.J. (2014). The apical actin fringe contributes to localized cell wall deposition and polarized growth in the lily pollen tube. Plant Physiol..

[30] Kost B., Lemichez E., Spielhofer P., Hong Y., Tolias K., Carpenter C., Chua N.H. (1999). Rac homologues and compartmentalized phosphatidylinositol 4, 5-bisphosphate act in a common pathway to regulate polar pollen tube growth. J. Cell Biol..

[31] Fu Y., Wu G., Yang Z. (2001). Rop GTPase-dependent dynamics of tip localized F-actin controls tip growth in pollen tubes. J. Cell Biol..

[32] Gu Y., Vernoud V., Fu Y., Yang Z. (2003). ROP GTPase regulation of pollen tube growth through the dynamics of tip-localized F-actin. J. Exp. Bot..

[33] Lee Y.J., Szumlanski A., Nielsen E., Yang Z. (2008). Rho-GTPase-dependent filamentous actin dynamics coordinate vesicle targeting and exocytosis during tip growth. J. Cell Biol..

[34] Smokvarska M., Jaillais Y., Martiniere A. (2021). Function of membrane domains in rho-of-plant signaling. Plant Physiol..

[35] Furt F., Konig S., Bessoule J.J., Sargueil F., Zallot R., Stanislas T., Noirot E., Lherminier J., Simon-Plas F., Heilmann I. (2010). Polyphosphoinositides are enriched in plant membrane rafts and form microdomains in the plasma membrane. Plant Physiol..

[36] Han B., Yang N., Pu H., Wang T. (2018). Quantitative proteomics and cytology of rice pollen sterol-rich membrane domains reveals pre-established cell polarity cues in mature pollen. J. Proteome.

[37] Lavy M., Bloch D., Hazak O., Gutman I., Poraty L., Sorek N., Sternberg H., Yalovsky S. (2007). A novel ROP/RAC effector links cell polarity, root-meristem maintenance, and vesicle trafficking. Curr. Biol..

[38] Li S., Gu Y., Yan A., Lord E., Yang Z.B. (2008). RIP1 (ROP Interactive Partner 1)/ICR1 marks pollen germination sites and may act in the ROP1 pathway in the control of polarized pollen growth. Mol. Plant.

[39] Del Casino C., Li Y.Q., Moscatelli A., Scali M., Tiezzi A., Cresti M. (1993). Distribution of microtubules during the growth of tobacco pollen tubes. Biol. Cell.

[40] Idilli A.I., Morandini P., Onelli E., Rodighiero S., Caccianiga M., Moscatelli A. (2013). Microtubule depolymerization affects endocytosis and exocytosis in the tip and influences endosome movement in tobacco pollen tubes. Mol. Plant.

[41] Astrom H. (1992). Acetylated alpha-tubulin in the pollen tube microtubules. Cell Biol. Int. Rep..

[42] Joos U., van Aken J., Kristen U. (1994). Microtubules are involved in maintaining the cellular polarity in pollen tubes of *Nicotiana sylvestris*. Protoplasma.

[43] Onelli E., Idilli A.I., Moscatelli A. (2015). Emerging roles for microtubules in angiosperm pollen tube growth highlight new research cues. Front. Plant Sci..

[44] Onelli E., Scali M., Caccianiga M., Stroppa N., Morandini P., Pavesi G., Moscatelli A. (2018). Microtubules play a role in trafficking prevacuolar compartments to vacuoles in tobacco pollen tubes. Open Biol..

[45] Nogales E., Wolf S.G., Downing K.H. (1998). Structure of the alpha beta tubulin dimer by electron crystallography. Nature.

[46] Nogales E. (2001). Structural insight into microtubule function. Annu. Rev. Biophys. Biomol. Struct..

[47] Mitchison T.K., Kirschner M. (1984). Dynamic instability of microtubule growth. Nature.

[48] Desai A., Mitchison T.J. (1997). Microtubule polymerization dynamics. Annu. Rev. Cell Dev. Biol..

[49] Horio T., Murata T. (2014). The role of dynamic instability in microtubule organization. Front. Plant Sci..

[50] Snustad D.P., Haas N.A., Kopczak S.D., Silflow C.D. (1992). The small genome of Arabidopsis contains at least nine expressed β-tubulin genes. Plant Cell.

[51] Carpenter J.L., Ploense S.E., Snustad D.P., Silflow C.D. (1992). Preferential expression of an α-tubulin gene of Arabidopsis in pollen. Plant Cell.

[52] Janke C., Bulinski J.C. (2011). Post-translational regulation of the microtubule cytoskeleton: Mechanisms and functions. Nat. Rev. Mol. Cell Biol..

[53] Gardiner J. (2019). Post-translational modifications of plant microtubules. Plant Sig. Behav..

[54] Chan J., Calder G.M., Doonan J.H., Lloyd C.W. (2003). EB1 reveals mobile microtubule nucleation sites in Arabidopsis. Nat. Cell Biol..

[55] Hamada T., Nagasaki-Takeuchi N., Kato T., Fujiwara M., Sonobe S., Fukao Y., Hashimoto T. (2013). Purification and characterization of novel microtubule-associated proteins from Arabidopsis cell suspension cultures. Plant Physiol..

[56] Hamada T. (2014). Microtubule organization and microtubule-associated proteins in plant cells. Int. Rev. Cell Mol. Biol..

[57] Goodson H.V., Jonasson E.M. (2018). Microtubules and microtubule-associated proteins. Cold Spring Harb. Perspect. Biol..

[58] Liu P., Würtz M., Zupa E., Pfeffer S., Schiebel E. (2021). Microtubule nucleation: The waltz between γ-tubulin ring complex and associated proteins. Curr. Opin. Cell Biol..

[59] Ehrhardt D.W., Shaw S.L. (2006). Microtubule dynamics and organization in the plant cortical array. Annu. Rev. Plant Biol..

[60] Ambrose J.C., Wasteneys G.O. (2008). CLASP modulates microtubule-cortex interaction during self-organization of acentrosomal microtubules. Mol. Biol. Cell.

[61] Elliott A., Shaw S.L. (2018). Update: Plant cortical microtubule arrays. Plant Physiol..

[62] Roll-Mecak A. (2020). The tubulin code in microtubule dynamics and information encoding. Dev. Cell.

[63] Lancelle S.A., Cresti M., Hepler P.K. (1987). Ultrastructure of the cytoskeleton in freeze-substituted pollen tubes of *Nicotiana alata*. Protoplasma.

[64] Cai G., Ovidi E., Romagnoli S., Vantard M., Cresti M., Tiezzi A. (2005). Identification and characterization of plasma membrane proteins that bind to microtubules in pollen tubes and generative cells of tobacco. Plant Cell Physiol..

[65] Schroeder R.J., Ahmed S.N., Zhu Y., London E., Brown D.A. (1998). Cholesterol and sphingolipid enhance the Triton X-100 insolubility of glycosylphosphatidylinositol-anchored proteins by promoting the formation of detergent-insoluble ordered membrane domains. J. Biol. Chem..

[66] London E., Brown D.A. (2000). Insolubility of lipids in triton X-100: Physical origin and relationship to sphingolipid/cholesterol membrane domains (rafts). Biochim. Biophys. Acta.

[67] Roche Y., Gerbeau-Pissot P., Buhot B., Thomas D., Bonneau L., Gresti J., Mongrand S., Perrier-Cornet J.M., Simon-Plas F. (2008). Depletion of phytosterols from the plant plasma membrane provides evidence for disruption of lipid rafts. FASEB J..

[68] Linetti A., Fratangeli A., Taverna E., Valnegri P., Francolini M., Cappello V., Matteoli M., Passafaro M., Rosa P. (2010). Cholesterol reduction impairs exocytosis of synaptic vesicles. J. Cell Sci..

[69] Villette C., Berna A., Compagnon V., Schaller H. (2015). Plant sterol diversity in pollen from angiosperms. Lipids.

[70] Stroppa N., Onelli E., Moreau P., Maneta-Peyret L., Berno V., Cammarota E., Ambrosini R., Caccianiga M.S., Scali M., Moscatelli A. (2023). Sterols and sphingolipids as new players in cell wall building and apical growth of *Nicotiana tabacum* L. pollen tubes. Plants.

[71] Repko E.M., Maltese W.A. (1989). Post-translational isoprenylation of cellular proteins is altered in response to mevalonate availability. J. Biol. Chem..

[72] Baxter A., Fitzgerald B.J., Hutson J.L., McCarthyS A.D., Motteram J.M., Ross B.C., Sapra M., Nowden M.A., Watson N.S., Williams R.J. (1992). Squalestatin 1, a potent inhibitor of squalene synthase, which lowers serum cholesterol in vivo. J. Biol. Chem..

[73] Bergstrom J.D., Kurtz M.M., Rew D.J., Amend A.M., Karkas J.D., Bostedor R.G., Bansal V.S., Dufresne C., Van Middlesworth F.L., Hensens O.D. (1993). Zaragozic acids: A family of fungal metabolites that are picomolar competitive inhibitors of squalene synthase. Proc. Natl. Acad. Sci. USA.

[74] Pechlivanis M., Kuhlmann J. (2006). Hydrophobic modifications of Ras proteins by isoprenoid groups and fatty acids—More than just membrane anchoring. Biochim. Biophys. Acta.

[75] Rotsch A.H., Kopka J., Feussner I., Ischebeck T. (2017). Central metabolite and sterol profiling divides tobacco male gametophyte development and pollen tube growth into eight metabolic phases. Plant J..

[76] Janke C., Magiera M.M. (2020). The tubulin code and its role in controlling microtubule properties and functions. Nature.

[77] Lefebvre B., Furt F., Hartmann M.A., Michaelson L.V., Carde J.P., Sargueil-Boiron F., Rossignol M., Napier J.A., Cullimore J., Bessoule J.J. (2007). Characterization of lipid rafts from *Medicago truncatula* root plasma membranes: A proteomic study reveals the presence of a raft-associated redox system. Plant Physiol..

[78] Goudenege S., Dargelos E., Claverol S., Bonneu M., Cottin P., Poussard S. (2007). Comparative proteomic analysis of myotube caveolae after milli-calpain deregulation. Proteomics.

[79] Whitehead S.N., Gangaraju S., Aylsworth A., Hou S.T. (2012). Membrane raft disruption results in neuritic retraction prior to neuronal death in cortical neurons. Bioscience Trends.

[80] Rodgers W., Zavzavadjian J. (2001). Glycolipid-enriched membrane domains are assembled into membrane patches by associating with the actin cytoskeleton. Exp. Cell Res..

[81] Douglass A.D., Vale R.D. (2005). Single-molecule microscopy reveals plasma membrane microdomains created by protein-protein networks that exclude or trap signaling molecules in T cells. Cell.

[82] Head B.P., Patel H.H., Roth D.M., Murray F., Swaney J.S., Niesman I.R., Farquhar M.G., Insel P.A. (2006). Microtubules and actin microfilaments regulate lipid raft/caveolae localization of adenylyl cyclase signaling components. J. Biol. Chem..

[83] Viola A., Gupta N. (2007). Tether and trap: Regulation of membrane-raft dynamics by actin-binding proteins. Nat. Rev. Immunol..

[84] Chichili G.R., Rodgers W. (2009). Cytoskeleton-membrane interactions in membrane raft structure. Cell. Mol. Life Sci..

[85] Suzuki T., Zhang J., Miyazawa S., Liu Q., Farzan M.R., Yao W.D. (2011). Association of membrane rafts and postsynaptic density: Proteomics, biochemical, and ultrastructural analyses. J. Neurochem..

[86] Ilangumaran S., Hoessli D.C. (1998). Effects of cholesterol depletion by cyclodextrin on the sphingolipid microdomains of the plasma membrane. Biochem. J..

[87] Tiwari S.C., Polito V.S. (1990). The initiation and organization of microtubules in germinating pear (*Pyrus communis* L.) pollen. Eur. J. Cell Biol..

[88] Hamada T., Ueda H., Kawase T., Hara-Nishimura I. (2014). Microtubules contribute to tubule elongation and anchoring of endoplasmic reticulum, resulting in high network complexity in Arabidopsis. Plant Physiol..

[89] Renna L., Stefano G., Slabaugh E., Wormsbaecher C., Sulpizio A., Zienkiewicz K., Brandizzi F. (2018). TGNap1 is required for microtubule-dependent homeostasis of a subpopulation of the plant trans-Golgi network. Nat. Commun..

[90] Delgadillo M.O., Ruano G., Zouhar J., Sauer M., Shen J., Lazarova A., Sanmartín M., Faat Lai L.T., Deng C., Wang P. (2020). MTV proteins unveil ER- and microtubule-associated compartments in the plant vacuolar trafficking pathway. Proc. Natl. Acad. Sci. USA.

[91] Lowe J., Li H., Downing K.H., Nogales E. (2001). Refined structure of alpha beta-tubulin at 3.5 A resolution. J. Mol. Biol..

[92] Stephens R.E. (1986). Membrane tubulin. Biol. Cell.

[93] Zambito A.M., Wolff J. (1997). Palmitoylation of tubulin. Biochem. Biophys. Res. Commun..

[94] Caron J.M. (1997). Posttranslational modification of tubulin by palmitoylation: I. in vivo and cell-free studies. Mol. Biol. Cell.

[95] Palestini P., Pitto M., Tedeschi G., Ferraretto A., Parenti M., Brunner J., Masserini M. (2000). Tubulin anchoring to glycolipid-enriched, detergent-resistant domains of the neuronal plasma membrane. J. Biol. Chem..

[96] Nakamura M., Ehrhardt D.W., Hashimoto T. (2010). Microtubule and katanin-dependent dynamics of microtubule nucleation complexes in the acentrosomal Arabidopsis cortical array. Nat. Cell Biol..

[97] Lindeboom J.J., Nakamura M., Marco Saltini M., Hibbel A., Walia A., Ketelaar T., Emons A.M.C., Sedbrook J.C., Kirik V., Mulder B.M. (2018). CLASP stabilization of plus ends created by severing promotes microtubule creation and reorientation. J. Cell Biol..

[98] Nakaoka Y., Akatsuki Kimura A., Tani T., Goshima G. (2015). Cytoplasmic nucleation and atypical branching nucleation generate endoplasmic microtubules in *Physcomitrella patens*. Plant Cell.

[99] Murata T., Sonobe S., Baskin T.I., Hyodo S., Hasezawa S., Nagata T., Horio T., Hasebe M. (2005). Microtubule-dependent microtubule nucleation based on recruitment of gamma-tubulin in higher plants. Nat. Cell Biol..

[100] Maekawa S., Kumanogoh H., Funatsu N., Takei N., Inoue K., Endo Y., Hamada K., Sokawa Y. (1997). Identification of NAP-22 and GAP-43 (neuromodulin) as major protein components in a Triton insoluble low density fraction of rat brain. Biochim. Biophys. Acta.

[101] Maekawa S., Morii H., Kumanogoh H., Sano M., Naruse Y., Sokawa Y., Mori N. (2001). Localization of neuronal growth-associated, microtubule-destabilizing factor SCG10 in brain-derived raft membrane microdomain. J. Biochem..

[102] Gardiner J. (2013). The evolution and diversification of plant microtubule associated proteins. Plant J..

[103] Tiezzi A., Moscatelli A., Cai G., Bartalesi A., Cresti M. (1992). An immunoreactive homolog of mammalian kinesin in *Nicotiana tabacum* pollen tube. Cell Motil. Cytoskel..

[104] Cai G., Bartalesi A., Del Casino C., Moscatelli A., Tiezzi A., Cresti M. (1993). The kinesin-immunoreactive homologue from *Nicotiana tabacum* pollen tubes: Biochemical properties and subcellular localization. Planta.

[105] Cai G., Romagnoli S., Moscatelli A., Ovidi E., Gambellini G., Tiezzi A., Cresti M. (2000). Identification and characterization of a novel microtubule-based motor associated with membranous organelles in tobacco pollen tubes. Plant Cell.

[106] Feiguelman G., Cui X., Sternberg H., Ben Hur E., Higa T., Oda Y., Fu Y., Yalovsky S. (2022). Microtubule-associated ROP interactors affect microtubule dynamics and modulate cell wall patterning and root hair growth. Development.

[107] Bratman S.V., Chang F. (2008). Mechanisms for maintaining microtubule bundles. Trends Cell Biol..

[108] Kong Z., Hotta T., Lee Y.R.J., Horio T., Liu B. (2010). The γ -tubulin complex protein GCP4 is required for organizing functional microtubule arrays in *Arabidopsis thaliana*. Plant Cell.

[109] Smertenko A.P., Chang H.-Y., Wagner V., Kaloriti D., Fenyk S., Sonobe S., Lloyd C., Hauser M.-T., Hussey P.J. (2004). The Arabidopsis microtubule-associated protein AtMAP65-1: Molecular analysis of its microtubule bundling activity. Plant Cell.

[110] Tulin A., McClerklin S., Huang Y., Dixit R. (2012). Single-molecule analysis of the microtubule cross-linking protein MAP65-1 reveals a molecular mechanism for contact-angle-dependent microtubule bundling. Biophys. J..

[111] Ambrose W.J.C., Shoji T., Kotzer A.M., Pighin J.A., Wasteneys G.O. (2007). The Arabidopsis CLASP gene encodes a microtubule-associated protein involved in cell expansion and division. Plant Cell.

[112] Thoms D., Vineyard L., Elliott A., Shaw S.L. (2018). CLASP facilitates transitions between cortical microtubule array patterns. Plant Physiol..

[113] Kirik V., Herrmann U., Parupalli C., Sedbrook J.C., Ehrhardt D.W., Hülskamp M. (2007). CLASP localizes in two discrete patterns on cortical microtubules and is required for cell morphogenesis and cell division in Arabidopsis. J. Cell Sci..

[114] Cai G., Moscatelli A., Del Casino C., Chevrier V., Mazzi M., Tiezzi A., Cresti M. (1996). The anti-centrosome monoclonal antibody 6C6 reacts with a plasma membrane-associated polypeptide of 77 kDa from *Nicotiana tabacum* pollen tubes. Protoplasma.

[115] Kollman J.M., Merdes A., Mourey L., Agard D.A. (2011). Microtubule nucleation by gamma-tubulin complexes. Nat. Rev. Mol. Cell Biol..

[116] Roostalu J., Surrey T. (2017). Microtubule nucleation: Beyond the template. Nat. Rev. Mol. Cell Biol..

[117] Keating T.J., Borisy G.G. (2000). Immunostructural evidence for the template mechanism of microtubule nucleation. Nat. Cell Biol..

[118] Stoppin V., Vantard M., Schmit A.C., Lambert A.M. (1994). Isolated plant nuclei nucleate microtubule assembly: The nuclear surface in higher plants has centrosome-like activity. Plant Cell.

[119] Erhardt M., Stoppin-Mellet V., Campagne S., Canaday J., Mutterer J., Fabian T., Sauter M., Muller T., Peter C., Lambert A.M. (2002). The plant Spc98p homologue colocalizes with γ-tubulin at microtubule nucleation sites and is required for microtubule nucleation. J. Cell Sci..

[120] Shaw S.L., Kamyar R., Ehrhardt D.W. (2003). Sustained microtubule treadmilling in Arabidopsis cortical arrays. Science.

[121] Van Bruaene N., Joss G., Van Oostveldt P. (2004). Reorganization and in vivo dynamics of microtubules during Arabidopsis root hair development. Plant Physiol..

[122] Efimov A., Kharitonov A., Efimova N., Loncarek J., Miller P.M., Andreyeva N., Gleeson P., Galjart N., Maia A.R., McLeod I.X. (2007). Asymmetric CLASP-dependent nucleation of noncentrosomal microtubules at the trans-Golgi Network. Dev. Cell.

[123] Luduena R.F. (1998). Multiple forms of tubulin: Different gene products and covalent modifications. Int. Rev. Cytol..

[124] Verhey K.J., Gaertig J. (2007). The tubulin code. Cell Cycle.

[125] Kumar N., Flavin M. (1981). Preferential action of a brain detyrosinolating carboxypeptidase on polymerized tubulin. J. Biol. Chem..

[126] Prota A.E., Magiera M.M., Kuijpers M., Bargsten K., Frey D., Wieser M., Jaussi R., Hoogenraad C.C., Kammerer R.A., Janke C. (2013). Structural basis of tubulin tyrosination by tubulin tyrosine ligase. J. Cell Biol..

[127] Kumar N., Flavin M. (1982). Modulation of some parameters of assembly of microtubules in vitro by tyrosinolation of tubulin. Eur. J. Biochem..

[128] Kreis T.E. (1987). Microtubules containing detyrosinated tubulin are less dynamic. EMBO J..

[129] Webster D.R., Gundersen G.G., Bulinski J.C., Borisy G.G. (1987). Differential turnover of tyrosinated and detyrosinated microtubules. Proc. Natl. Acad. Sci. USA.

[130] Cambray-Deakin M.A., Burgoyne R.D. (1987). Acetylated and detyrosinated alpha- tubulins are co- localized in stable microtubules in rat meningeal fibroblasts. Cell Motil. Cytoskel..

[131] Erck C., Peris L., Andrieux A., Meissirel C., Gruber A.D., Vernet M., Schweitzer A., Saoudi Y., Pointu H., Bosc C. (2005). A vital role of tubulin-tyrosine-ligase for neuronal organization. Proc. Natl. Acad. Sci. USA.

[132] Peris L., Thery M., Fauré J., Saoudi Y., Lafanechère L., Chilton J.K., Gordon-Weeks P., Galjart N., Bornens M., Wordeman L. (2006). Tubulin tyrosination is a major factor affecting the recruitment of CAP- Gly proteins at microtubule plus ends. J. Cell Biol..

[133] Peris L., Wagenbach M., Lafanechere L., Brocard J., Moore A.T., Kozielski F., Job D., Wordeman L., Andrieux A. (2009). Motor-dependent microtubule disassembly driven by tubulin tyrosination. J. Cell Biol..

[134] Sirajuddin M., Rice L.M., Vale R.D. (2014). Regulation of microtubule motors by tubulin isotypes and post-translational modifications. Nat. Cell Biol..

[135] Eddé B., Rossier J., Le Carer J.P., Desbruyères E., Gros F., Denoulet P. (1990). Posttranslational glutamylation of alpha-tubulin. Science.

[136] Audebert S., Desbruyères E., Gruszczynski C., Koulakoff A., Gros F., Denoulet P., Eddé B. (1993). Reversible polyglutamylation of alpha- and beta- tubulin and microtubule dynamics in mouse brain neurons. Mol. Biol. Cell.

[137] Janke C., Rogowski K., Wloga D., Regnard C., Kajava A.V., Strub J.M., Temurak N., van Dijk J., Boucher D., van Dorsselaer A. (2005). Tubulin polyglutamylase enzymes are members of the TTL domain protein family. Science.

[138] Rogowski K., van Dijk J., Magiera M.M., Bosc C., Deloulme J.C., Bosson A., Peris L., Gold N.D., Lacroix B., Bosch Grau M. (2010). A family of protein-deglutamylating enzymes associated with neurodegeneration. Cell.

[139] Wolff A., Houdayer M., Chillet D., de Ne´chaud B., Denoulet P. (1994). Structure of the polyglutamyl chain of tubulin: Occurrence of alpha and gamma linkages between glutamyl units revealed by monoreactive polyclonal antibodies. Biol. Cell.

[140] Song Y., Kirkpatrick L.L., Schilling A.B., Helseth D.L., Chabot N., Keillor J.W., Johnson G.V.W., Brady S. (2013). Transglutaminase and polyamination of tubulin: Posttranslational modification for stabilizing axonal microtubules. Neuron.

[141] Bonnet C., Boucher D., Lazereg S., Pedrotti B., Islam K., Denoulet P., Larcher J.C. (2001). Differential binding regulation of microtubule-associated proteins MAP1A, MAP1B, and MAP2 by tubulin polyglutamylation. J. Biol. Chem..

[142] Brewbaker J.L., Kwack B.H. (1963). The essential role of calcium ions in pollen germination and pollen tube growth. Am. J. Bot..

[143] Schneider C.A., Rasband W.S., Eliceiri K.W. (2012). NIH Image to ImageJ: 25 years of image analysis. Nat. Methods.

[144] Laemmli U.K. (1970). Cleavage of structural proteins during the assembly of the head of bacteriophage T4. Nature.

[145] Braford M.M. (1976). A rapid and sensitive method for the quantitation of microgram quantities of protein utilizing the principle of protein-dye binding. Anal. Biochem..

[146] Sinha P., Poland J., Schnolzer M., Rabilloud T. (2001). A new silver staining apparatus and procedure for matrix-assisted laser desorption/ionization-time of flight analysis of proteins after two-dimensional electrophoresis. Proteomics.

[147] Towbin H., Staehelin T., Gordon J. (1979). Electrophoretic transfer of proteins from polyacrylamide gels to nitrocellulose sheets. Procedure and some applications. Proc. Natl. Acad. Sci. USA.

[148] Heilemann M., van de Linde S., Schüttpelz M., Kasper R., Seefeldt B., Mukherjee A., Tinnefeld P., Sauer M. (2008). Subdiffraction-resolution fluorescence imaging with conventional fluorescent probes. Angew. Chem. Int. Ed..

[149] Frangi A.F., Niessen W.J., Hoogeveen R.M., van Walsum T., Viergever M.A. (1999). Model-based quantitation of 3-D magnetic resonance angiographic images. IEEE Trans. Med. Imaging.

[150] Jerman T. (2022). Jerman Enhancement Filter. GitHub. https://github.com/timjerman/JermanEnhancementFilter.

[151] Bolte S., Cordelières F.P. (2006). A guided tour into subcellular colocalization analysis in light microscopy. J. Microsc..

