# Supplementary material for: Inhibition of Sterol Biosynthesis Alters Tubulin Association with Detergent-Insoluble Membranes and Affects Microtubule Organization in Pollen Tubes of Nicotiana tabacum L
Source: Plants (Basel). 2025 Dec 17;14(24):3845. doi: 10.3390/plants14243845 (PMC12736560; doi:10.3390/plants14243845)
Supplement: Supplementary file 1 [file plants-14-03845-s001.zip › plants-3989656-supplementary.pdf]

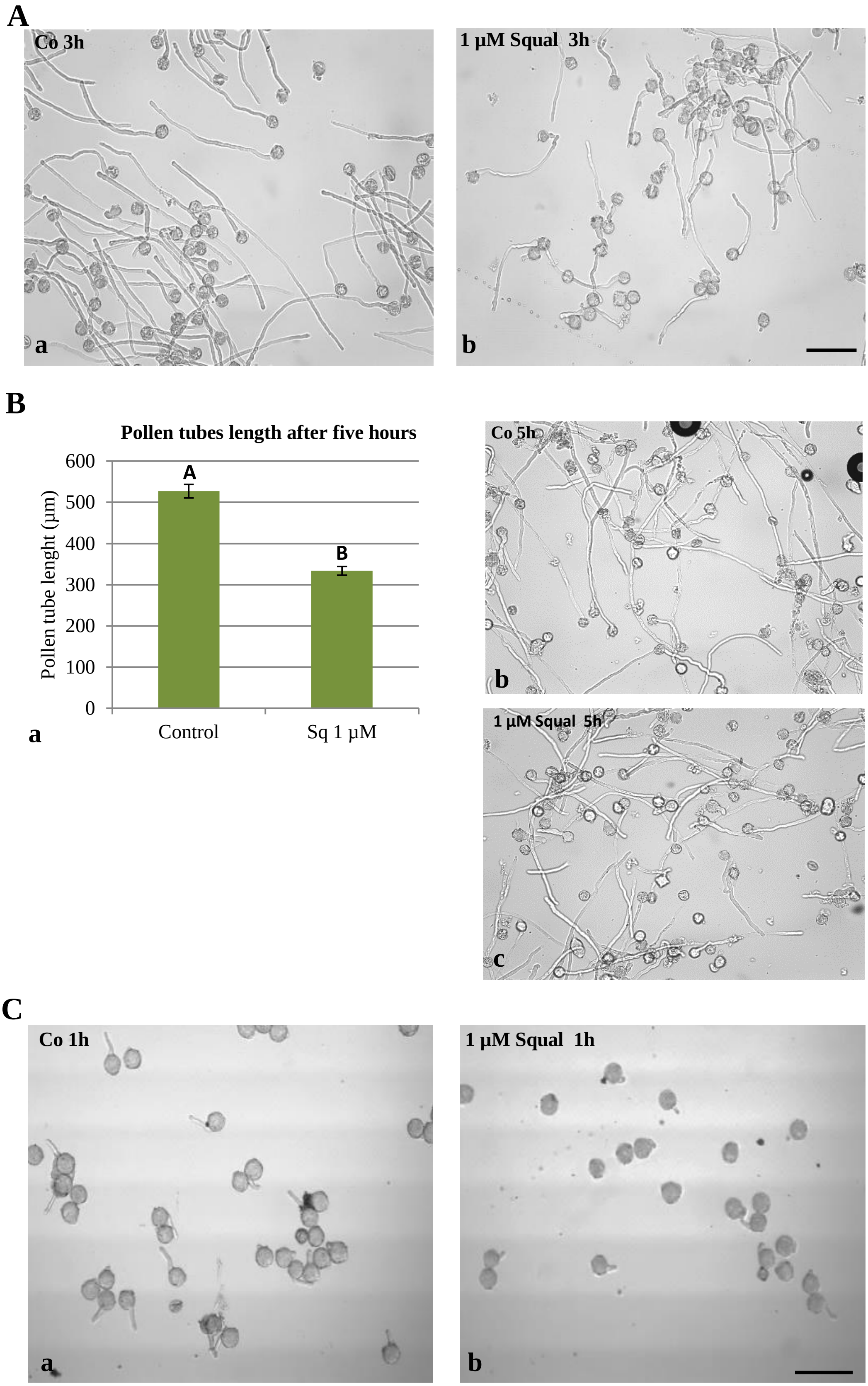
A
Co 3h
a
1 µM Squal 3h
b
B
Pollen tubes length after five hours
Pollen tube lenght (µm)
600
500
400
300
200
100
0
A
B
Control
Sq 1 µM
a
Co 5h
b
1 µM Squal 5h
c
C
Co 1h
a
1 µM Squal 1h
b

Fig. S1

Supplementary Figure 1. Effect of squalestatin on pollen tube growth. (A) Pollen tubes were grown for 3 hours with or without 1 μM Sq. Squalestatin-treated pollen tubes were considerably shorter (b) than control tubes (a). (B) Pollen tubes grown for 5 hours with 1 μM Sq were still significantly shorter than controls (a-c; Student's t test $p<0.001$). (C) Germination assay experiments showed that 1 μM Sq delayed pollen germination and pollen tube emission (a, b). Bar=100 μm.

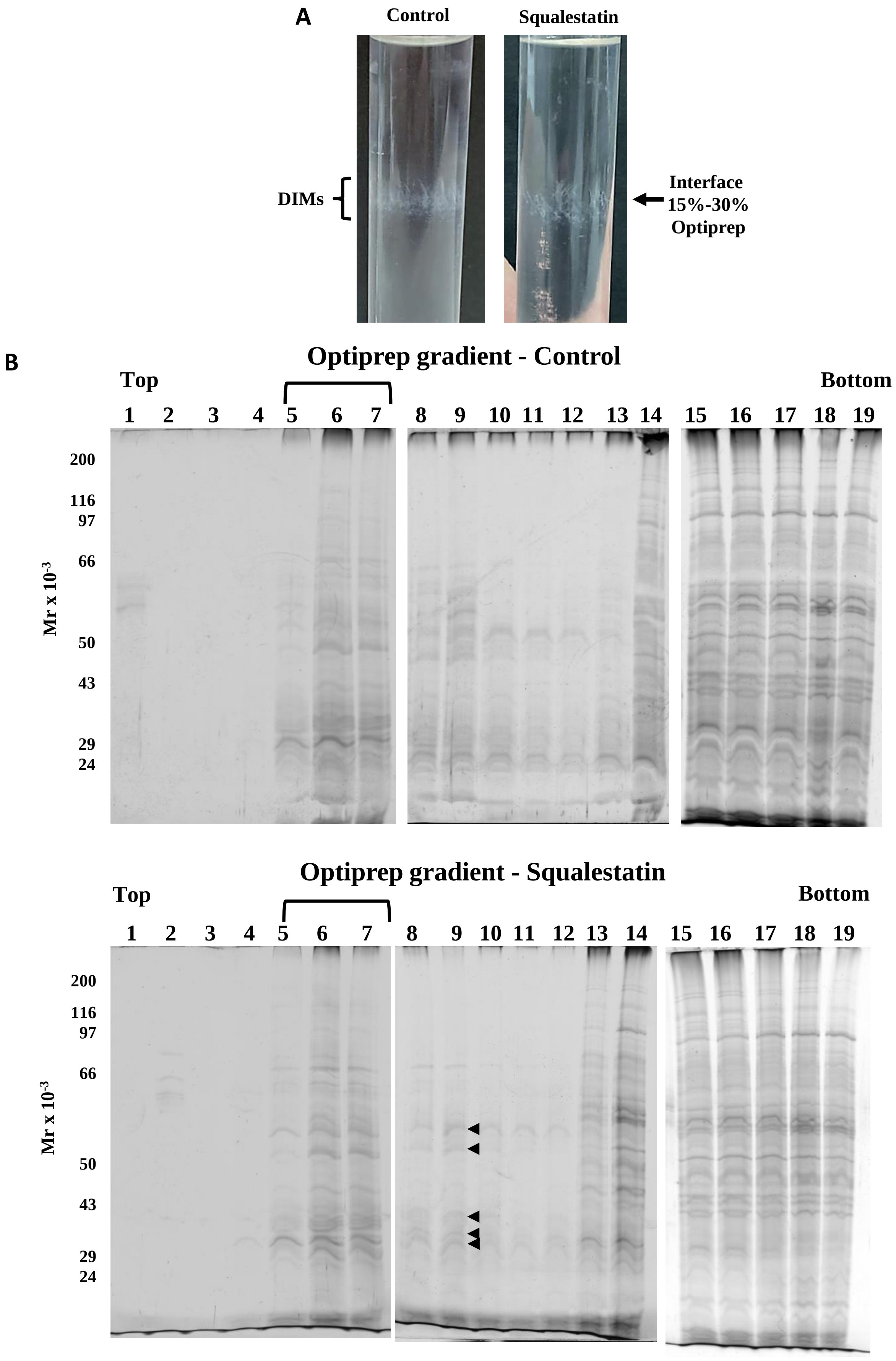
A
Control
Squalestatin
DIMs
Interface
15%-30%
Optiprep
B
Optiprep gradient - Control
Top
Bottom
1 2 3 4 5 6 7 8 9 10 11 12 13 14 15 16 17 18 19
200
116
97
66
50
43
29
24
Mr x 10-3
Optiprep gradient - Squalestatin
Top
Bottom
1 2 3 4 5 6 7 8 9 10 11 12 13 14 15 16 17 18 19
200
116
97
66
50
43
29
24
Mr x 10-3

Fig.S2

Supplementary Figure 2. Optiprep gradients. (A) Images of control and squalestatin Optiprep gradients. In both cases DIMs were recovered as floating material at the interface between 15% and 30% Optiprep. However, while control DIMs appeared to consist of finely particulate material, Sq-DIMs had a lamellar appearance. (B) Electrophoretic profile (SDS-PAGE) of fractions derived from the Optiprep gradients (control/squalestatin). Squalestatin gradient fractions 8 and 9 showed a smaller amount of polypeptides than control and a different electrophoretic profile. In particular, only two polypeptides with molecular mass between 50 and 66 kDa and three polypeptides with molecular mass between 43 and 29 kDa were visible in fraction 9 (arrowheads).

In control gradient the number of polypeptides decreased in fractions 10-13, while fraction 14 showed many polypeptides with molecular mass from 116 to 24 kDa. While control fraction 13 showed few faint bands, Sq gradient fraction 13 revealed a greater number of polypeptides, ranging in mass from 200 to 29 kDa. Fraction 14 showed a large number of polypeptides in both samples but the electrophoretic profiles appeared to be different.

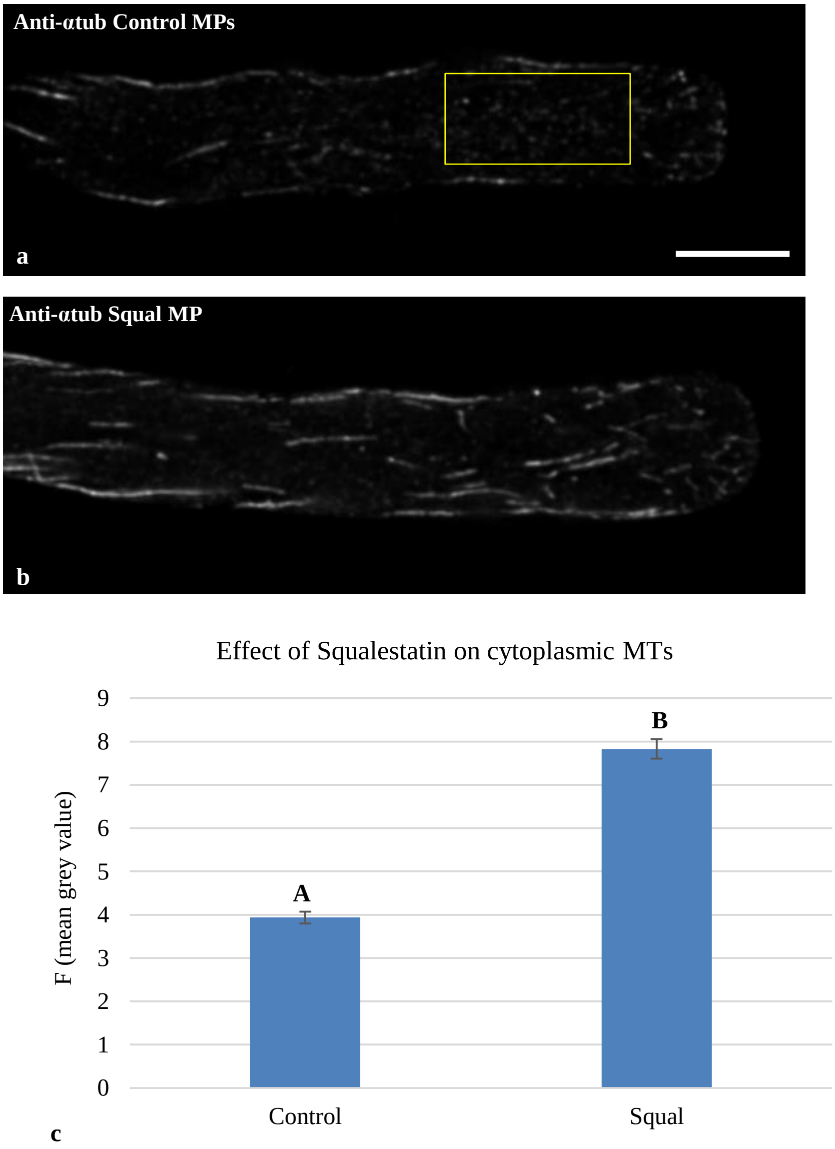

Fig. S3

Supplementary Figure 3. Effect of squalestatin on cytoplasmic MTs. (a) Observation of shank medial planes in control pollen tubes revealed sparse short MT segments. (b) Quite long MTs were observed in the corresponding regions of Sq-treated pollen tubes. (c) Quantification analysis of mean fluorescence in five medial stacks for each pollen tube (ImageJ, multi measure option) showed significantly more cytoplasmic MTs in the shank of Sq-treated pollen tubes than in controls (Student's test $p<0.05$, $n\geq16$). Scale bar=10 μm.

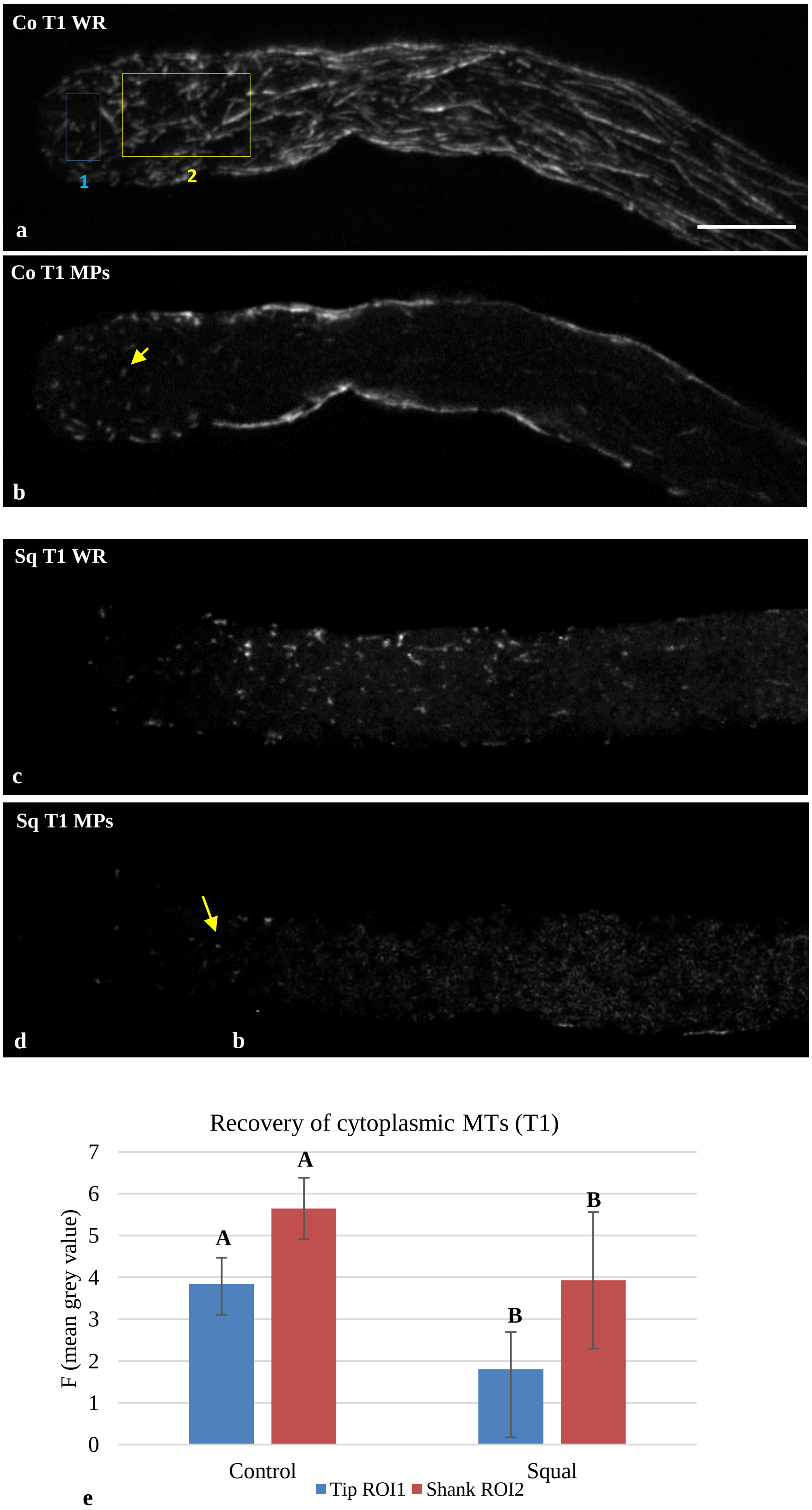
Co T1 WR
1
2
a
Co T1 MPs
b
Sq T1 WR
c
Sq T1 MPs
d
b
Recovery of cytoplasmic MTs (T1)
F (mean grey value)
7
6
5
4
3
2
1
0
A
A
B
B
Control
Squal
Tip ROI1
Shank ROI2
e

Fig.S4

Supplementary Figure 4. Recovery of cytoplasmic MTs after oryzalin washout. (a, b) Cortical MTs recovered at T1 along the pollen tube showed the typical distribution in control cells (a, WR) with short MT bundles in the shank (a, WR yellow ROI2; b, blue arrows) and rare short MTs in the tip (a, WR Blue ROI1; b). Observation of medial stacks at T1 (ImageJ, multi measure option on five stacks) showed very short MT fragments and tubulin spots rather than cytoplasmic MTs (b MPs, MPs; yellow arrow). (c, d) In Sq-treated pollen tubes only rare MT spots were observed in the cytoplasm (d MPs, yellow arrow). (e, f) Statistical analysis of mean fluorescence intensity showed that recovery of cytoplasmic MTs was similar in the tip and the shank both in control and Squaestatin samples (a, e blue ROI1 and yellow ROI2; Two-way ANOVA and Post-hoc Tukey test $p>0.05$. N-Co=23, N-Sq = 20). Two-way ANOVA test also showed that the recovery in the Squalestatin samples was significantly less efficient than in the control (Post-hoc Tukey test ROI1 Co versus ROI1 Sq p=2,15E-06; ROI2 Co versus ROI2 Sq p=0,003347). Scale bar=10 µm.
